# Pyroptosis–Ferroptosis Crosstalk Suggests Candidate Molecular Clusters and Immune Remodeling in Peri-Implantitis

**DOI:** 10.3390/genes17060664

**Published:** 2026-06-07

**Authors:** Xinda Li, Zhijia Liu, Jiaxuan Nie, Jianing Wang, Jinlai Bao, Wuwei Li

**Affiliations:** 1 Department of Oral and Maxillofacial Surgery, School of Stomatology, Dalian Medical University, Dalian 116044, China; 2 School of Stomatology, Dalian Medical University, Dalian 116044, China; 3 School of Stomatology, Changsha Medical University, Changsha 410219, China; 4 School of Stomatology, Dalian University, Dalian 116622, China

**Keywords:** peri-implantitis, pyroptosis, ferroptosis, candidate molecular clusters, immune microenvironment, BRAF, TRPV1

## Abstract

**Background:** Peri-implantitis is a major biological complication for the long-term stability of dental implants but its molecular heterogeneity and mechanism of programmed cell death are unknown. The present study aimed to elucidate the molecular characteristics and alterations in the immune microenvironment of peri-implantitis from the perspective of pyroptosis–ferroptosis crosstalk. **Methods:** We retrospectively integrated three public GEO transcriptomic datasets (GSE178351, GSE33774, and GSE57631), comprising 29 peri-implant tissue samples, including 16 peri-implantitis samples and 13 healthy controls. Batch effects were corrected, followed by differential expression analysis, GSEA, GSVA, consensus clustering, machine learning-based exploratory feature prioritization, immune-infiltration estimation, and predicted ceRNA network construction. **Results:** A total of 2450 disease-associated candidate differentially expressed genes were identified, among which 41 genes were associated with both pyroptosis and ferroptosis. Pathway analysis indicated significant upregulation of inflammatory responses, complement activation, TNF-α/NF-κB, IL-6/JAK/STAT3, reactive oxygen species (ROS) pathways and tissue remodeling-related processes in peri-implantitis tissues. Based on these 41 overlap genes, unsupervised clustering suggested two candidate expression clusters, C1 and C2, in the integrated cohort. C1 was predominantly composed of peri-implantitis samples and showed stronger inflammatory and cellular-stress-related pathway activity. *BRAF* and *TRPV1* were prioritized as exploratory candidate genes and showed associations with estimated immune-infiltration patterns. **Conclusions:** This exploratory analysis suggests that pyroptosis–ferroptosis crosstalk-related signatures may be associated with immune remodeling in peri-implantitis. *BRAF* and *TRPV1* may serve as candidate genes for future validation, while the C1/C2 clusters should be interpreted as preliminary expression patterns rather than established disease subtypes.

## 1. Background

Peri-implantitis is one of the major biological complications that jeopardize the long-term stability of dental implants, and it is characterized by inflammation of the soft tissues surrounding the implant and progressive marginal bone loss [1,2]. In addition to plaque-related biofilm accumulation, peri-implantitis and implant failure may also be influenced by patient-related factors, local tissue conditions, surgical or prosthetic factors, and systemic inflammatory or metabolic status [2]. Current clinical diagnosis is primarily based on phenotypic indicators like probing depth, bleeding on probing, pus discharge, and radiographic bone resorption [3]. These indicators are mainly a measure of structural damage that has already occurred and therefore are not easily able to reveal disease activity and possible molecular heterogeneity in a timely manner [4]. In recent years, there has been a growing body of research on molecular biomarkers in saliva and peri-implant sulcus fluid [5]. Peri-implantitis has been associated with IL-1β, IL-6, TNF-α, matrix metalloproteinases, and oxidative stress markers such as GSH-Px, MPO, and MDA [6]. However, the results from different studies are still inconsistent and the standardization of testing and external validation are relatively underwhelming [7]. Consequently, a stable and generalizable molecular stratification and risk identification system for peri-implantitis is still missing.

From a pathophysiological perspective, peri-implantitis is not a linear inflammatory process triggered solely by plaque accumulation, but rather the result of the combined effects of biofilm dysbiosis, abnormal host immune responses, cumulative oxidative stress, and bone metabolism disorders [2]. Current research shows that peri-implantitis tissue has different immunological microenvironment features as compared to healthy peri-implant tissue and periodontitis tissue, such as increased immune-cell infiltration, changed matrix cell composition, and increased inflammatory responses [8]. These findings suggest that peri-implantitis is not a homogeneous disease but rather consists of distinct tissue, immunological and molecular states [9]. Previous studies have mainly focused on individual inflammatory biomarkers, broad transcriptomic alterations, or immune-cell composition in peri-implantitis. However, it remains unclear whether regulated cell death-related signatures, particularly pyroptosis–ferroptosis crosstalk, can be used to characterize disease-associated transcriptional patterns and immune microenvironment remodeling in an integrated framework.

Programmed cell death provides a new perspective for understanding the amplification of inflammation and tissue destruction in peri-implantitis [10]. Pyroptosis is a programmed cell death with inflammation, which is featured by the disruption of cell membrane integrity and the release of pro-inflammatory factors including IL-1β and IL-18 [11]. Ferroptosis is defined as iron-dependent lipid peroxidation and causes damage to membrane lipids [12]. Both processes are closely related to inflammation, oxidative stress and tissue damage and have been involved in the pathogenesis and progression of periodontal and other oral inflammatory diseases [11,13]. In peri-implantitis lesions, persistent biofilm stimulation, inflammatory cytokine release, oxidative stress, and progressive tissue destruction may coexist within the same local microenvironment. Therefore, pyroptosis- and ferroptosis-related signatures may represent interconnected inflammatory and oxidative stress responses rather than isolated cell death programs [2,9]. However, systematic studies on the co-dysregulation of molecules associated with pyroptosis and ferroptosis and their capacity to characterize disease-related candidate expression clusters and mirror changes in the immune microenvironment are still deficient in peri-implantitis [7,14]. Thus, the primary aim of this study was to explore pyroptosis–ferroptosis crosstalk-related transcriptional signatures in peri-implantitis using integrated public transcriptomic datasets. The secondary aims were to characterize related pathway activity, identify candidate molecular clusters, estimate immune-infiltration patterns, and prioritize candidate genes for future validation.

## 2. Method

### 2.1. Data Acquisition and Preprocessing

In this study, three gene expression microarray datasets related to peri-implantitis tissues were downloaded from the Genome Expression Omnibus (GEO) at the National Center for Biotechnology Information (NCBI), namely GSE178351, GSE33774 and GSE57631. Datasets were included if they met the following criteria: human peri-implant tissue samples, available disease and healthy control groups, accessible normalized or raw expression profiles, and identifiable sample grouping information. Samples without clear disease/control annotation or genes without reliable annotation were excluded from downstream analysis. The datasets consist of 29 samples in total, with 16 samples of peri-implantitis tissue and 13 samples of healthy peri-implant tissue. The available information mainly included dataset source, platform annotation, sample grouping, and disease/control status. However, detailed patient-level clinical variables, etiological factors, microbiological profiles, implant characteristics, treatment history, and surgical or prosthetic information were not consistently available across the three datasets. Therefore, these variables could not be incorporated as covariates in the integrated differential expression model. The raw expression matrices were background corrected and quantile normalized. In case of multiple probes corresponding to the same gene symbol, the maximum expression value was chosen to avoid redundancy. The three datasets were then batch effect corrected by using the ComBat algorithm in the sva R package. The correction effect was visually assessed by principal component analysis (PCA), which showed that batch effects were effectively removed.

### 2.2. Screening for Exploratory Candidate Differentially Expressed Genes

We used linear models implemented in the limma R package together with empirical Bayes moderation to compare gene expression differences between peri-implantitis and healthy control samples. Differential expression analysis was performed using the ComBat-corrected expression matrix. Considering the limited sample size and the exploratory nature of this integrated transcriptomic analysis, candidate differentially expressed genes were initially screened using an exploratory threshold of Benjamini–Hochberg adjusted *p* value < 0.30 and |log2 fold change| ≥ 0.15. This threshold was used to retain potentially relevant transcriptional signals for downstream hypothesis-generating analyses rather than to define definitive disease-driving genes [15]. To evaluate the influence of this exploratory threshold, sensitivity analyses were further performed using more stringent criteria, including nominal *p* value < 0.05, adjusted *p* value < 0.25, and adjusted *p* value < 0.05, all combined with |log2 fold change| ≥ 0.15. The threshold-based sensitivity analysis is summarized in Supplementary Table S5.

### 2.3. Acquisition of Pyroptosis and Ferroptosis-Related Gene Sets

Pyroptosis-related genes were collected from MSigDB/GSEA, literature-curated gene lists, and GeneCards keyword searches. Ferroptosis-related genes were obtained from MSigDB/GSEA, the literature-curated gene lists, GeneCards, and the KEGG ferroptosis pathway [16]. GeneCards was searched on 8 March 2026 using the keywords ‘pyroptosis’ and ‘ferroptosis’, and only genes with a relevance score ≥1.2 were retained. All gene symbols were harmonized to official gene symbols and duplicates were removed before downstream analysis. The sources and final non-redundant gene lists are provided in Supplementary Tables S1–S4. Because GeneCards keyword searches may include genes with indirect or relatively weak associations, the resulting gene sets were used as exploratory candidate gene sets rather than definitive pathway-specific gene lists.

### 2.4. Gene Set Enrichment Analysis

Gene Set Enrichment Analysis (GSEA) was performed to determine the differences in pathway activity between the peri-implantitis group and the healthy control group for the whole transcriptomic level. The reference database was the HALLMARK annotated gene sets (v2023.2) from the Molecular Signatures Database (MSigDB). All identified genes were ranked in descending order of their log2 fold change (log2FC) and used as a pre-ranked list input into the clusterProfiler R package for GSEA [17]. Multiple hypothesis testing correction was performed using the Benjamini–Hochberg method and pathways with false discovery rate (FDR) < 0.25 were defined as significantly enriched pathways.

### 2.5. Gene Set Variation Analysis

To assess pathway activity at the single-sample level, GSVA was employed to generate pathway enrichment scores for each sample. Enrichment scores of each pathway in each sample were calculated by the single-sample gene set enrichment analysis (ssGSEA) algorithm of the GSVA R package, with the MSigDB HALLMARK gene sets (v2023.2) as reference annotations [18]. The difference in pathway activity between the peri-implantitis group and healthy control group was then compared by empirical Bayesian method in limma R package, with correction for multiple hypothesis testing by Benjamini–Hochberg. GSVA computes pathway activity at the single-sample level, thus effectively accounting for heterogeneity between samples.

### 2.6. Consensus Clustering for Candidate Molecular Clusters

To explore the existence of candidate expression clusters of peri-implantitis based on genes involved in pyroptosis–ferroptosis crosstalk, consensus clustering was performed with the expression matrix of the 41 pyroptosis–ferroptosis overlap genes identified in the previous screening. Data were centered by the median of each gene before clustering. Unsupervised clustering was performed using the hierarchical clustering algorithm with the Pearson correlation distance as the distance metric, using the ConsensusClusterPlus R package [19]. The parameters were as follows: To evaluate clustering stability, 200 resampling iterations were performed, with 80% of samples and 80% of gene features randomly selected in each iteration. The consensus matrix, cumulative distribution function (CDF) curve, delta area plot, and tracking plot for K = 2 to K = 6 were used to evaluate clustering stability and to select the optimal cluster number. To confirm the degree of separation among samples of different subtypes, PCA scatter plots were used after candidate expression clusters were identified. Fisher’s exact test was used to assess the association between the candidate cluster classification and clinical status (disease/health).

To compare the differences in pathway activity between the two candidate expression clusters, GSVA was used to generate 50 ssGSEA enrichment scores of HALLMARK pathways for each sample. Differences in pathway activity between the two candidate clusters were compared by the empirical Bayesian method in the limma R package, with an FDR < 0.05 as the criterion of statistical significance. The top 30 differentially expressed pathways were ranked by t-values. Wilcoxon rank-sum test was used to determine the significance of the expression differences in the 41 pyroptosis–ferroptosis overlap genes between the two candidate clusters, with *p* < 0.05 as the cutoff for differentially expressed genes.

### 2.7. Machine Learning-Based Exploratory Feature Prioritization

Two complementary machine learning feature selection strategies were used to find the minimal set of exploratory candidate features of the pyroptosis–ferroptosis overlap genes among the 41 pyroptosis–ferroptosis overlap genes. First, the dimensionality of the features was reduced using LASSO (Least Absolute Shrinkage and Selection Operator) regression. The regularization parameter was selected by 10-fold cross-validation, and genes with non-zero coefficients at λ1se were retained as LASSO-selected candidates. Secondly, the Support Vector Machine with Recursive Feature Elimination (SVM-RFE) algorithm was employed. The genes with the smallest contribution to the classification were iteratively removed using 10-fold cross-validation and the accuracy with different feature numbers was recorded to select the optimal feature subset. The last core exploratory candidate genes were determined by the intersection of genes screened by LASSO and SVM-RFE. A multivariate logistic regression model was established for combined exploratory model, with the final pyroptosis–ferroptosis overlap genes as independent variables and disease status as the dependent variable. Receiver operator characteristic (ROC) curves were plotted and the area under the curve (AUC) was calculated to determine internal discriminatory performance. The intersection of the LASSO- and SVM-RFE-selected genes was defined as the exploratory candidate feature set. Because of the limited sample size, the machine learning workflow was used for exploratory feature prioritization rather than for establishing a clinically validated diagnostic model. The initial ROC analysis was retained as an apparent/internal performance estimate in the integrated cohort. To reduce the instability introduced by a single 7:3 split, leave-one-out cross-validation (LOOCV) and repeated five-fold cross-validation with 100 repetitions were further performed for BRAF, TRPV1, and the combined BRAF + TRPV1 logistic model. AUC values and 95% confidence intervals were calculated where applicable, and the results were interpreted as exploratory internal performance.

### 2.8. Estimation of Immune-Infiltration Patterns

CIBERSORT was applied to the batch-corrected expression matrix to estimate the relative proportions of 22 LM22-defined leukocyte subsets in each sample. The LM22 signature matrix was used as the reference, and deconvolution was performed with 1000 permutations [20]. For each sample, the CIBERSORT *p* value, correlation coefficient, and RMSE were recorded as quality metrics. Among the 29 samples, 19 had a CIBERSORT *p* value < 0.05. Considering the small sample size and the exploratory purpose of this analysis, all samples were retained for downstream immune-infiltration comparisons. Differences in immune-cell fractions between groups were assessed using the Wilcoxon rank-sum test, and Benjamini–Hochberg correction was applied across the 22 immune-cell types. The results were interpreted as estimated leukocyte-infiltration patterns rather than complete cellular composition profiles of peri-implant tissues.

### 2.9. Construction of a Predicted ceRNA Network

To explore potential post-transcriptional regulatory hypotheses related to BRAF and TRPV1, a predicted competing endogenous RNA (ceRNA) network was constructed. Candidate miRNAs targeting BRAF and TRPV1 were retrieved from miRTarBase (https://mirtarbase.cuhk.edu.cn (accessed on 18 April 2026)) and starBase (https://starbase.sysu.edu.cn (accessed on 18 April 2026)). Candidate lncRNAs interacting with these miRNAs were then obtained from lncBase (https://diana.e-ce.uth.gr/ (accessed on 18 April 2026)) and starBase. An lncRNA–miRNA–mRNA regulatory network was constructed and visualized using the igraph R package. Because matched lncRNA and miRNA expression profiles were not available in the included peri-implantitis datasets, co-expression or inverse-expression relationships could not be evaluated. Therefore, this ceRNA network was interpreted as a database-derived computational prediction rather than evidence of active post-transcriptional regulation in peri-implantitis tissues.

### 2.10. Statistical Analysis

All computational analyses were performed using R software 4.4.3. Differential expression analysis was conducted with the limma package, batch correction with the sva package, functional enrichment and GSEA with clusterProfiler and related Bioconductor packages, GSVA with the GSVA package, consensus clustering with ConsensusClusterPlus, machine learning-based feature prioritization with glmnet and e1071/caret, immune deconvolution with the CIBERSORT R script and LM22 signature matrix, and network visualization with igraph. Fisher’s exact test was used to evaluate the association between candidate cluster assignment and disease status. Spearman correlation analysis was used to assess relationships between candidate gene expression and estimated immune-cell fractions. As this study was based exclusively on public transcriptomic datasets and computational analysis, no laboratory hardware, sampling equipment, or experimental instruments were used.

## 3. Results

### 3.1. Identification of Exploratory Candidate Differentially Expressed Genes and Pyroptosis–Ferroptosis Overlap Genes

**Figure 1:**
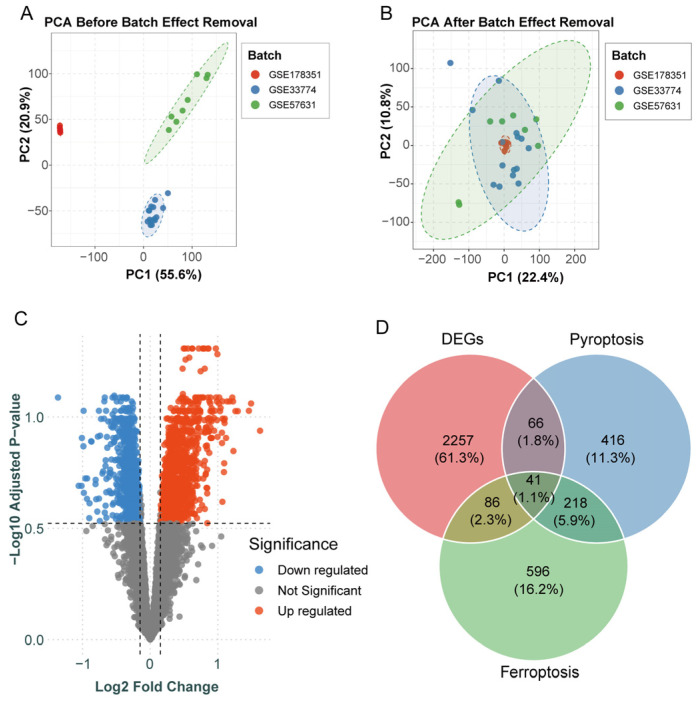
Multi-cohort data integration, differential expression analysis, and screening of pyroptosis–ferroptosis overlap genes in peri-implantitis. (**A**). Principal component analysis (PCA) before batch effect correction shows that samples from different datasets are clearly separated by batch. (**B**). PCA after batch effect correction shows that samples from each dataset are fully mixed, indicating successful integration. (**C**). Volcano plot showing exploratory candidate differentially expressed genes between peri-implantitis and healthy control samples. The volcano plot was generated using Benjamini–Hochberg adjusted *p* value and log2 fold-change thresholds; the exploratory screening threshold was adjusted *p* value < 0.30 and |log2FC| ≥ 0.15. (**D**). Venn diagram of differentially expressed genes (DEGs), pyroptosis-related genes, and ferroptosis-related genes; the intersection yields 41 candidate genes at the pyroptosis–ferroptosis overlap.

In the present study, three transcriptome datasets related to peri-implantitis (GSE178351, GSE33774, and GSE57631) were integrated, comprising a total of 29 tissue samples, including 16 peri-implantitis samples and 13 healthy peri-implant tissue samples. After integrated gene annotation, expression matrix processing and ComBat batch effect correction, the PCA results showed that the separation of samples caused by different dataset sources was significantly decreased compared with pre-correction, meaning that the integrated expression matrix can be used for subsequent analysis (Figure 1A,B).

We compared the transcriptional differences between the peri-implantitis group and the healthy control group using the corrected expression matrix. Under exploratory screening thresholds, we identified a total of 2450 disease-associated candidate differentially expressed genes (Figure 1C). Sensitivity analyses using stricter thresholds yielded progressively fewer candidate genes, supporting the interpretation of this DEG set as an exploratory candidate pool rather than a definitive disease-driving gene set. The threshold-based sensitivity analysis is summarized in Supplementary Table S5. Further intersection analysis of the candidate differentially expressed genes with the pyroptosis-related gene set and ferroptosis-related gene set ultimately identified 41 candidate genes simultaneously belonging to the differentially expressed gene set, pyroptosis gene set and ferroptosis gene set (Figure 1D). These genes were defined as pyroptosis–ferroptosis crossover candidate genes associated with peri-implantitis, and used for subsequent pathway analysis, molecular subtyping and screening for candidate exploratory candidate genes.

### 3.2. GSEA Reveals Significant Activation of Inflammation, Complement, and Oxidative Stress-Related Pathways in Peri-Implantitis

**Figure 2:**
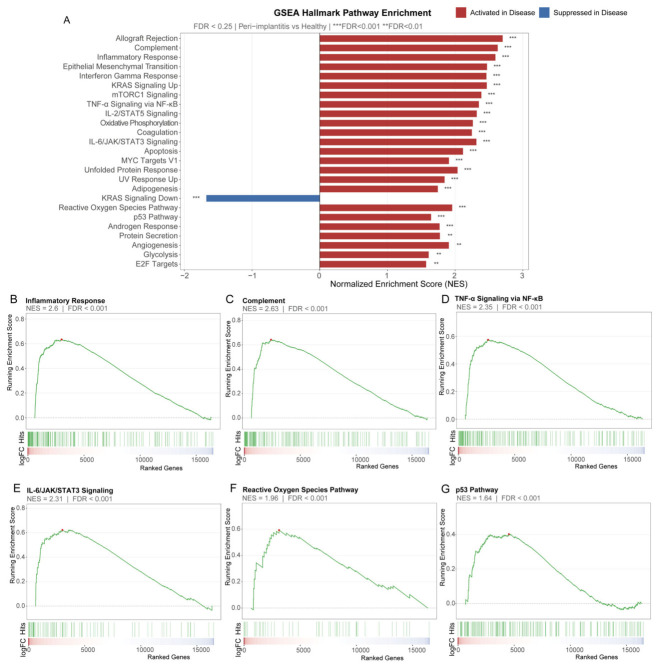
GSEA reveals significant activation of inflammation, complement, and oxidative stress-related pathways in peri-implantitis. (**A**). Overview of GSEA pathway enrichment based on the HALLMARK gene set. Positive NES indicates that the pathway is enriched in the peri-implantitis group, while negative NES indicates that the pathway is relatively enriched in the healthy control group. (**B**–**G**). Representative GSEA enrichment plots show that inflammatory response, complement, NF-κB-mediated TNF-α signaling, IL-6/JAK/STAT3 signaling, reactive oxygen species (ROS) signaling, and p53 signaling are all significantly positively enriched in peri-implantitis tissues.

Pathway enrichment analysis indicated significant enrichment of peri-implantitis tissue in several immune, inflammatory and cellular stress-related pathways, including allograft rejection, complement, inflammatory response, epithelial–mesenchymal transition, interferon-gamma response, TNF-α signaling via NF-κB, IL-6/JAK/STAT3 signaling, apoptosis, reactive oxygen species pathway and p53 pathway (Figure 2A). Among the representative pathways, Inflammatory response (NES = 2.60, FDR < 0.001) and Complement (NES = 2.63, FDR < 0.001) were significantly positively enriched (Figure 2B,C). Meanwhile, TNF-α signaling via NF-κB (NES = 2.35, FDR < 0.001), IL-6/JAK/STAT3 signaling (NES = 2.31, FDR < 0.001) and the reactive oxygen species pathway (NES = 1.96, FDR < 0.001) were also significantly enriched in the disease group (Figure 2D–F). The results suggest that peri-implantitis is associated not only with increased local inflammatory responses but also with complement activation, increased cytokine signaling and upregulation of oxidative stress-related pathways. These pathways characteristics are consistent with previously identified pyroptosis–ferroptosis crossover candidate genes, providing a functional basis for further analysis of the molecular heterogeneity of the disease.

### 3.3. GSVA Further Confirms Enhanced Activity of Inflammation, Oxidative Stress, and Tissue Remodeling Pathways in Peri-Implantitis

**Figure 3:**
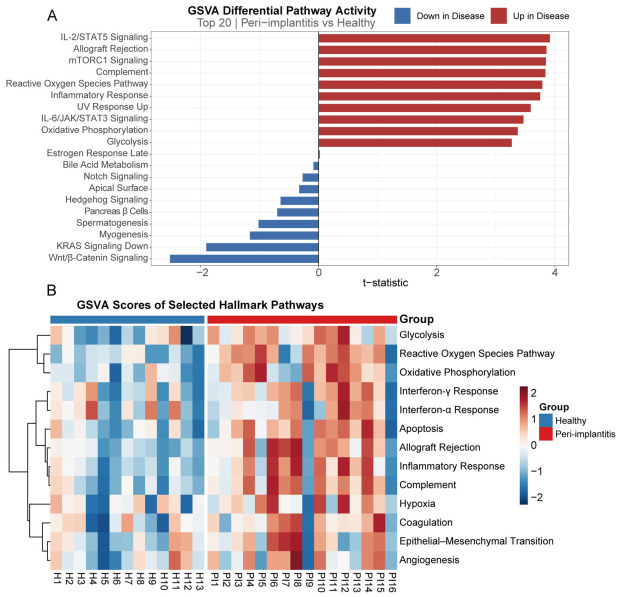
GSVA reveals pathway activity remodeling at the sample level in peri-implantitis. (**A**). Bar charts showing differential activity of representative HALLMARK pathways based on GSVA scores. (**B**). Heatmap of GSVA scores for HALLMARK pathways related to inflammation, oxidative stress, cellular stress, and tissue remodeling.

Samples from peri-implantitis had higher activity scores for several immune–inflammatory, oxidative stress, and tissue remodeling pathways, including Inflammatory Response, Complement, IL-6/JAK/STAT3 Signaling, Reactive Oxygen Species Pathway, mTORC1 Signaling, Oxidative Phosphorylation, and Glycolysis (Figure 3A). Additional targeted analysis found that peri-implantitis samples had higher overall pathway scores for Reactive Oxygen Species Pathway, Inflammatory Response, Complement, Apoptosis, Hypoxia, Epithelial–Mesenchymal Transition, and Angiogenesis (Figure 3B). These findings support the GSEA findings at a single-sample level, suggesting a certain degree of heterogeneity in pathway activity in peri-implantitis samples, which provides the foundation for subsequent molecular subtyping based on key genes involved in pyroptosis and ferroptosis. The complete GSEA and GSVA pathway-level statistics are provided in Supplementary Tables S6 and S7.

### 3.4. Identification of Candidate Expression Clusters Associated with Peri-Implantitis Based on Pyroptosis–Ferroptosis Overlap Genes

**Figure 4:**
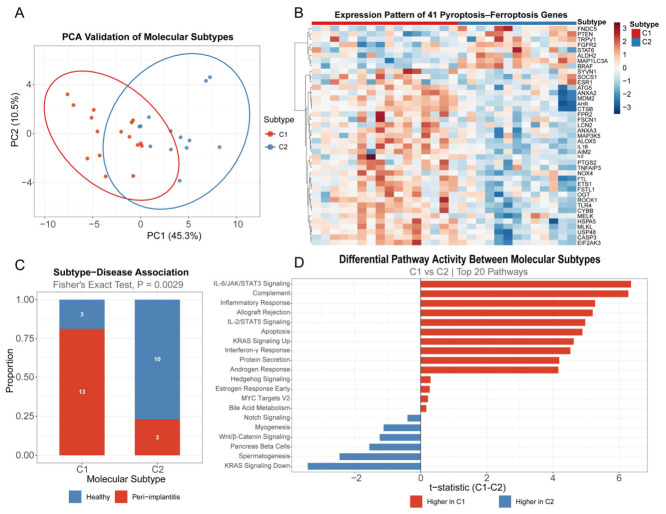
Candidate expression clusters associated with peri-implantitis based on pyroptosis–ferroptosis overlap genes. (**A**). PCA plot based on the expression profiles of the 41 pyroptosis–ferroptosis overlap genes. C1 and C2 showed a tendency to separate in the principal component space, with PC1 and PC2 explaining 45.3% and 10.5% of the total variance, respectively. (**B**). Heatmap showing the expression patterns of the 41 pyroptosis–ferroptosis overlap genes in C1 and C2. (**C**). Distribution of healthy controls and peri-implantitis samples in the C1 and C2 candidate expression clusters. C1 contained 13 peri-implantitis samples and three healthy control samples, whereas C2 contained three peri-implantitis samples and 10 healthy control samples. Fisher’s exact test was used to assess the association between cluster assignment and disease status. (**D**). Bar chart showing HALLMARK pathway activity differences between C1 and C2. C1 and C2 should be interpreted as candidate expression clusters rather than established molecular subtypes of peri-implantitis.

Based on the expression matrix of the 41 pyroptosis–ferroptosis overlap genes, consensus clustering was performed to explore candidate expression clusters in the integrated cohort. Joint evaluation of the consensus matrix, CDF curve, delta area plot, and tracking plot supported K = 2 as the optimal clustering solution (Figure S1). The two resulting candidate expression clusters were named C1 and C2 (Figure 4A,B). The PCA analysis on the expression profiles of these 41 genes showed that C1 and C2 were well-separated in the principal component space with PC1 and PC2 explaining 45.3% and 10.5% of the total variance, respectively (Figure 4A).

Further analysis of the relationship between subtypes and disease status revealed that C1 comprised mostly peri-implantitis samples and C2 comprised mostly healthy control samples. Specifically, C1 contained 13 peri-implantitis samples and three healthy samples, while C2 contained three peri-implantitis samples and 10 healthy samples. Fisher’s exact test showed a significant association between candidate expression clusters and disease status (*p* = 0.0029, Figure 4C). This result suggests that the candidate expression clusters identified according to the pyroptosis–ferroptosis overlap genes are not random expression clusters, but are closely related to the disease status of peri-implantitis.

GSVA analysis showed significant differences in immune inflammation, cellular stress, and programmed cell death pathways between C1 and C2, including IL-6/JAK/STAT3 signaling, Complement, Inflammatory response, Allograft rejection, IL-2/STAT5 signaling and Apoptosis (Figure 4D). In addition, the 41 pyroptosis–ferroptosis overlap genes showed different expression patterns in the two subtypes. Some genes associated with inflammation, oxidative stress and cell death were relatively up-regulated in C1, while TRPV1, BRAF, MAP1LC3A and PTEN genes were relatively up-regulated in C2 (Figure 4D).

In summary, two candidate expression clusters with distinct expression patterns, disease distributions, and pathway activity characteristics were identified by the unsupervised clustering of 41 pyroptosis–ferroptosis overlap genes. Subtype C1, with more prominent molecular features of inflammation and cellular stress, was significantly associated with peri-implantitis. This suggests that peri-implantitis is not a single homogeneous inflammatory state but may consist of specific molecular subpopulations driven by pyroptosis–ferroptosis-related genes.

### 3.5. Exploratory Prioritization of BRAF and TRPV1 as Candidate Genes Associated with Pyroptosis–Ferroptosis Crosstalk

**Figure 5:**
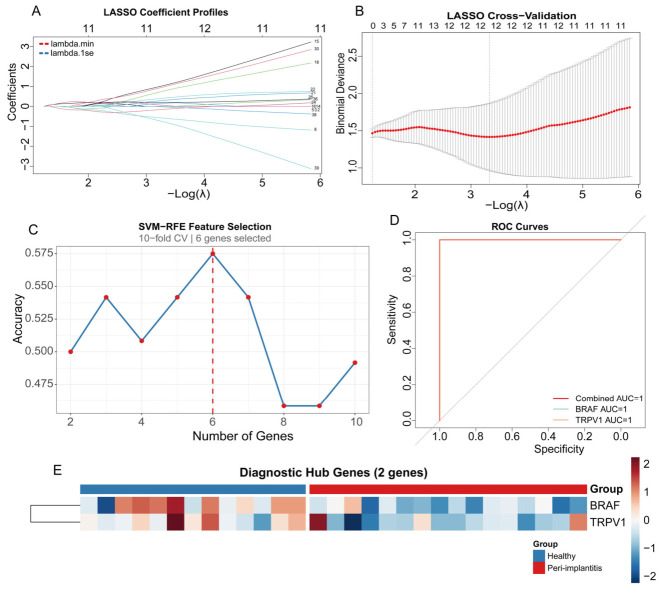
Exploratory prioritization of *BRAF* and *TRPV1* as candidate genes associated with pyroptosis–ferroptosis crosstalk. (**A**). LASSO regression coefficient path plot showing changes in the coefficients of candidate genes under different regularization parameters. (**B**). LASSO cross-validation curve used to determine the optimal regularization parameter and screen candidate genes. (**C**). SVM-RFE feature selection curve showing the model’s cross-validation accuracy for different numbers of features. (**D**). ROC curves for *BRAF*, *TRPV1*, and the combined *BRAF* + *TRPV1* model in the integrated cohort of 29 samples, including 16 peri-implantitis samples and 13 healthy controls. These ROC curves indicate apparent/internal performance only and should not be interpreted as validated diagnostic performance. (**E**). Expression patterns of *BRAF* and *TRPV1* in healthy controls and peri-implantitis samples. ROC curves indicate apparent/internal performance only; LOOCV and repeated five-fold cross-validation results are provided in Supplementarys Table S8 and Figure S2.

As the value of the regularization coefficient increases, the coefficient path plot of LASSO shows that the coefficients of candidate genes gradually shrink, indicating that this method can effectively reduce redundant features (Figure 5A). With the regularization parameters from cross-validation, LASSO finally selected 12 candidate genes for further analysis (Figure 5B). In the SVM-RFE algorithm, 41 pyroptosis–ferroptosis overlap genes were ranked by recursively removing features with low classification contribution. The results of 10-fold cross-validation show that the model has relatively optimal performance when the number of features is six (Figure 5C). Two common candidate genes, BRAF and TRPV1, were identified by the intersection of LASSO and SVM-RFE screening results. The expression heatmaps show a clear expression pattern of *BRAF* and *TRPV1* in healthy controls and peri-implantitis samples (Figure 5E). The intersection of the LASSO and SVM-RFE results retained two genes, *BRAF* and *TRPV1*, as exploratory candidate features. The ROC curves shown in Figure 5 represent apparent/internal performance within the integrated cohort and were not interpreted as validated diagnostic performance. To further address the potential instability of a single data split, LOOCV and repeated five-fold cross-validation were performed. In LOOCV, the AUC values were 0.774 for *BRAF*, 0.721 for *TRPV1*, and 0.745 for the combined *BRAF* + *TRPV1* model. Repeated five-fold cross-validation showed mean AUC values of 0.789, 0.761, and 0.776, respectively. These findings indicate moderate internal discriminatory performance but should be interpreted as exploratory feature prioritization results rather than evidence of clinical diagnostic utility (Supplementarys Table S8 and Figure S2).

### 3.6. Estimated Immune-Infiltration Patterns in Peri-Implantitis and Candidate Expression Clusters

The CIBERSORT algorithm was used to estimate the relative proportions of 22 LM22-defined leukocyte subsets in the integrated cohort. Among the 29 samples, 19 passed the CIBERSORT significance threshold of *p* value < 0.05. Group comparisons between peri-implantitis and healthy control samples were performed using the Wilcoxon rank-sum test, followed by Benjamini–Hochberg correction across the 22 immune-cell types. Although monocytes and CD8 T cells showed nominal differences between groups, no immune-cell subset remained significant after FDR correction (Supplementary Table S9). Therefore, the immune-infiltration results were interpreted as exploratory trends rather than definitive differences in tissue cellular composition.

**Figure 6:**
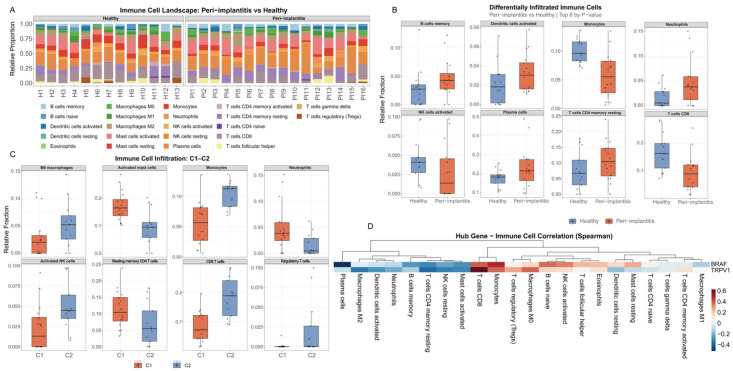
Estimated immune-infiltration patterns in peri-implantitis and candidate expression clusters. (**A**). A stacked bar chart comparing the immune-cell profiles of the Healthy group and the Peri-implantitis group, showing differences in the relative proportions of various immune-cell subsets between the two groups. (**B**). CIBERSORT analysis showing the top 8 immune-cell types with the most significant differences between healthy controls and peri-implantitis samples. Each point represents a sample; box plots indicate the median and interquartile range. (**C**). The top-eight immune-cell types with the representative immune-cell subsets ranked by nominal *p* values between the C1 and C2 candidate expression clusters. (**D**). Spearman correlation heatmap between BRAF and TRPV1 expression levels and the relative infiltration proportions of 22 immune-cell types. *p* values for immune-cell comparisons were calculated using the Wilcoxon rank-sum test, and Benjamini–Hochberg correction was applied across the 22 immune-cell types. Exact nominal and adjusted *p* values are provided in Supplementary Table S9.

Based on nominal *p* values and descriptive differences, peri-implantitis samples tended to show relatively higher proportions of neutrophils, activated dendritic cells, plasma cells, and resting memory CD4 T cells, together with relatively lower proportions of CD8 T cells, activated NK cells, and monocytes (Figure 6A,B). These patterns are consistent with an inflammatory and immune-remodeled local microenvironment, but should be interpreted cautiously because of the limited sample size and the exploratory nature of deconvolution analysis. Immune-infiltration patterns were further compared between the C1 and C2 candidate expression clusters. Compared with C2, C1 showed relatively higher estimated levels of neutrophils, activated mast cells, and resting memory CD4 T cells, whereas C2 showed relatively higher estimated levels of M0 macrophages, monocytes, activated NK cells, CD8 T cells, and regulatory T cells (Figure 6C). Because C1 was predominantly composed of peri-implantitis samples, these differences may partly reflect disease-associated immune changes rather than independent subtype-specific immune characteristics. In addition, Spearman correlation analysis suggested associations between *BRAF*/*TRPV1* expression and selected immune-cell fractions (Figure 6D). Overall, these results suggest that peri-implantitis and its candidate expression clusters may be accompanied by altered estimated immune-infiltration patterns, while further validation using larger cohorts, single-cell analysis, or histological approaches is required.

### 3.7. Construction of a Predicted ceRNA Regulatory Network Centered on BRAF and TRPV1

**Figure 7:**
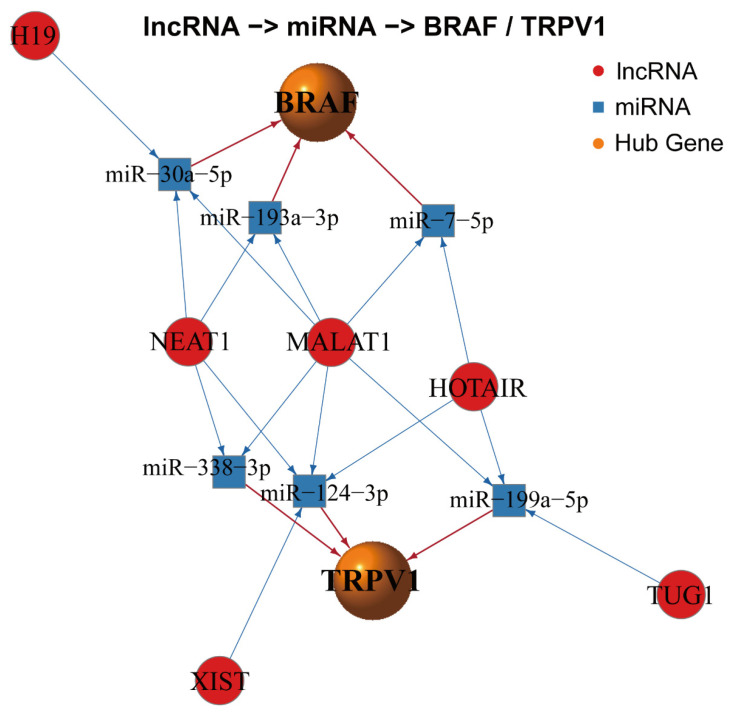
Predicted ceRNA network centered on *BRAF* and *TRPV1*. This network was constructed based on public interaction databases and should be interpreted as a computational prediction rather than a validated regulatory mechanism in peri-implantitis tissues.

To explore potential post-transcriptional regulatory hypotheses related to *BRAF* and *TRPV1*, a database-derived predicted ceRNA network was constructed using public interaction resources, including miRTarBase, starBase, and lncBase. The network contained three types of nodes: lncRNAs, miRNAs, and mRNAs, including six lncRNAs, six miRNAs, and two exploratory candidate genes. Specifically, the lncRNA nodes included NEAT1, MALAT1, H19, HOTAIR, TUG1, and XIST; the miRNA nodes included miR-30a-5p, miR-193a-3p, miR-7-5p, miR-199a-5p, miR-338-3p, and miR-124-3p; and the mRNA nodes were BRAF and TRPV1 (Figure 7).

This network suggested several possible lncRNA–miRNA–mRNA regulatory axes involving *BRAF* and *TRPV1*. However, because matched lncRNA and miRNA expression profiles were not available in the included peri-implantitis datasets, these predicted interactions could not be validated by co-expression or inverse-expression analysis. Therefore, the ceRNA network should be interpreted as a computational hypothesis for future validation rather than evidence of active post-transcriptional regulation in peri-implantitis tissues.

## 4. Discussion

This study systematically analyzed disease-associated transcriptional abnormalities, pathway activation remodeling, candidate expression clusters, immune-infiltration characteristics and candidate exploratory candidate genes by integrating peri-implantitis transcriptomic data based on the crosstalk between pyroptosis and ferroptosis. Taken together, this study demonstrated an exaggerated classical inflammatory response in peri-implantitis tissues, but also the co-dysregulation of genes involved in complement activation, oxidative stress, cellular stress and programmed cell death. Previous studies have demonstrated that IL-1β, IL-6, TNF-α, MMP-8, RANKL and other markers in gingival crevicular fluid or saliva are associated with peri-implantitis [6,21]. Wang et al. report that peri-implant disease activity is also associated with local oxidative stress markers like GSH-Px, MPO and MDA, suggesting that oxidative stress could be an important link between inflammatory responses and tissue destruction [22]. Unlike these studies, the present study did not focus on single oxidative stress markers or individual inflammatory factors, but proposed a molecular framework at the whole-transcriptome level, involving the joint participation of inflammation, oxidative stress and programmed cell death. Peri-implantitis should be understood as a multifactorial condition rather than a disease driven by a single cause [2]. Patient-related factors, plaque control, local peri-implant tissue conditions, implant positioning, prosthetic design, biomechanical loading, systemic metabolic status and microbial dysbiosis may all contribute to disease initiation and progression [8,21]. In this context, the transcriptomic changes observed in the present study should be interpreted as downstream host-response patterns associated with peri-implantitis tissues, rather than gene expression changes attributable to one specific etiological factor.

GSEA and GSVA results indicated that TNF-α/NF-κB, IL-6/JAK/STAT3, Complement, Inflammatory response and Reactive oxygen species pathways were significantly enriched in peri-implantitis tissue. These findings largely corroborate previous studies that have identified immune dysregulation, complement activation and heightened inflammatory pathways in peri-implantitis [8,23]. Programmed cell death offers new insight to understand this complex inflammatory state [24]. Pyroptosis can amplify the local inflammatory responses through inflammatory caspase, Gasdermin family proteins and IL-1β/IL-18 release [25,26]. In contrast, ferroptosis is closely related to iron homeostasis disorder, lipid peroxidation and membrane lipid damage [12]. Xu et al. reported that pyroptosis may connect microbial stimulation, inflammasome activation, and the release of immune factors to promote periodontal tissue destruction, and ferroptosis may also occur under the stimulation of plaque microorganisms and the inflammatory microenvironment and contribute to damaging periodontal hard and soft tissues [27]. Tang et al. studied ferroptosis in bone-associated cells and proposed that iron metabolism, mitochondrial function, and the balance of bone formation and bone resorption could be influenced by inflammatory factors and oxidative stress [28]. In this study, we further included genes related to pyroptosis and ferroptosis in the analysis simultaneously and identified 41 overlapping candidate genes. It suggests that these two cell death-related programs may be jointly disrupted against the backdrop of inflammation and oxidative stress in peri-implantitis.

We performed consensus clustering based on 41 pyroptosis–ferroptosis overlap genes and observed two candidate expression clusters, C1 and C2, which were significantly associated with disease status. Interestingly, subtype C1 was enriched in peri-implantitis samples and subtype C2 was enriched in healthy controls, indicating that this clustering is not a random expression cluster but may reflect disease-specific transcriptional patterns. Li et al. found by single-cell analysis that peri-implantitis has a unique immune microenvironment different from healthy peri-implant tissues and periodontitis tissues, with increased immune-cell infiltration, altered stromal cell composition, and increased inflammatory responses [8]. Because this study is based on bulk transcriptomics, it is impossible to determine cell origins of the subtypes at single-cell level. However, the C1/C2 classification should be interpreted cautiously. Because clustering was performed in the integrated cohort containing both peri-implantitis and healthy control samples, the separation may mainly reflect disease-associated transcriptional differences rather than true intra-disease candidate expression clusters of peri-implantitis. Therefore, C1 and C2 should be regarded as preliminary candidate expression clusters that require validation in larger peri-implantitis-only cohorts [29].

In this study, LASSO and SVM-RFE were simultaneously used to screen *BRAF* and *TRPV1* as candidate exploratory candidate genes for pyroptosis and ferroptosis. *TRPV1*-positive sensory neurons were shown to participate in alveolar bone resorption and the regulation of bone metabolism via CGRP signaling in animal studies by Takahashi et al. [30]. In addition, the study by Jiang et al. on dental implants also suggests that *TRPV1* activation in sensory neurons modulates macrophage polarization and immune responses through CGRP release, which facilitates osseointegration [31]. Thus, *TRPV1* may connect neuro-immune regulation, bone remodeling and peri-implant tissue homeostasis. According to the NCBI gene annotations, *BRAF* encodes an RAF family serine/threonine protein kinase, which has been reported to be involved in MAPK/ERK signaling and MAPK-related inflammatory pathways in the immune response of peri-implantitis [32,33]. *BRAF* is also known as an oncology-related gene involved in MAPK/ERK signaling [34]. However, the present study analyzed BRAF expression in non-tumor peri-implantitis datasets and did not assess *BRAF* mutations or malignant transformation. Therefore, our findings should not be interpreted as evidence of a cancer-related mechanism in peri-implantitis, but rather as a possible link to MAPK-related inflammatory signaling that requires further validation. Nevertheless, the evidence in the present study is based on integrated bulk transcriptomic analysis and internal feature prioritization. Because of the limited sample size and lack of independent external validation, protein-level confirmation, or functional experiments, *BRAF* and *TRPV1* should be interpreted as exploratory candidate genes rather than validated clinical diagnostic biomarkers.

The predicted ceRNA network provided a computational hypothesis regarding potential post-transcriptional regulation involving *BRAF* and *TRPV1*. This study predicts that lncRNAs such as NEAT1, MALAT1, H19, HOTAIR, TUG1 and XIST may form regulatory networks with *BRAF*/*TRPV1* through associated miRNAs. Previous studies have proposed that lncRNAs, such as MALAT1 and NEAT1, may be involved in the regulation of inflammatory responses and cellular functions in periodontitis and other oral inflammatory diseases [35,36]. However, the ceRNA network constructed in this study is all predicted from the public databases, and no lncRNA or miRNA expression matrix has been used to validate the ceRNA network. Thus, the role of this network is rather to generate testable mechanistic hypotheses than to prove the existence of specific lncRNA–miRNA–mRNA regulatory axes. Clinically, these findings may provide preliminary molecular clues for future risk stratification and disease-activity assessment in peri-implantitis. However, they are not currently applicable as diagnostic or therapeutic tools and require validation in larger prospective cohorts.

Some limitations exist in this study. First, all analyses were based on publicly available microarray datasets with a small sample size. Although batch correction was performed, dataset heterogeneity and possible residual batch effects cannot be completely excluded. In addition, detailed clinical metadata, etiological factors, microbiological profiles, implant characteristics and treatment history were not consistently available, which limited further covariate-adjusted analyses. The diagnostic utility of BRAF and TRPV1 and the stability of the C1/C2 candidate expression clusters still need to be validated in larger clinical cohorts. Second, the differential expression analysis employed an exploratory screening threshold to retain candidate genes that could be functionally relevant in the context of chronic inflammation, but this could also increase the risk of false-positive findings. Third, CIBERSORT results are computational estimates of relative leukocyte proportions and cannot resolve spatial distribution, cell–cell interactions, rare cell populations, or non-immune tissue components; therefore, histological, immunohistochemical, single-cell, or spatial transcriptomic validation is required. Fourth, this study did not incorporate microbiome, proteomic or metabolomic data, although these omics dimensions are important for understanding the links among biofilm dysbiosis, host responses and bone destruction. Fifth, the candidate genes and predicted ceRNA network have not been experimentally validated, and the specific regulatory relationships need to be further confirmed by protein-level assays, dual-luciferase reporter assays, RNA interference, overexpression experiments, and models using cells derived from peri-implantitis tissues.

## 5. Conclusions

This exploratory integrated transcriptomic analysis suggests that peri-implantitis is associated with inflammatory response, complement activation, oxidative stress, tissue remodeling, and regulated cell death-related transcriptional changes. By integrating pyroptosis- and ferroptosis-related gene sets, we identified 41 overlap genes and observed two candidate expression clusters associated with disease status. *BRAF* and *TRPV1* emerged as exploratory candidate genes linked to these transcriptional and immune-infiltration patterns. These findings should be regarded as hypothesis-generating and require validation in larger independent cohorts, clinically annotated samples, and experimental models.

## Data Availability

The transcriptomic datasets analyzed in this study are publicly available in the GEO database, and the accession numbers are provided in the manuscript. The curated pyroptosis-related and ferroptosis-related gene sets used in this study are provided in the Supplementary Materials. All other data generated or analyzed during this study are included in this published article and its supplementary files. If further information is required, it can be obtained from the corresponding author upon reasonable request.

## Supplementary Materials

The following supporting information can be downloaded at https://www.mdpi.com/article/10.3390/genes17060664/s1, Figure S1. Consensus clustering evaluation based on the 41 pyroptosis–ferroptosis overlap genes; Figure S2. LOOCV ROC curves for exploratory candidate features; Table S1. Sources of pyroptosis-related genes; Table S2. Final non-redundant pyroptosis-related gene set; Table S3. Sources of ferroptosis-related genes; Table S4. Final non-redundant ferroptosis-related gene set; Table S5. Sensitivity analysis of differential expression thresholds; Table S6. Complete GSEA pathway-level statistics; Table S7. Complete GSVA differential pathway statistics; Table S8. Internal validation of BRAF, TRPV1, and the combined model as exploratory candidate features; Table S9. Immune-cell comparisons between peri-implantitis and healthy control samples with multiple-testing correction.
