# Supplementary material for: Pyroptosis–Ferroptosis Crosstalk Suggests Candidate Molecular Clusters and Immune Remodeling in Peri-Implantitis
Source: Genes (Basel). 2026 Jun 7;17(6):664. doi: 10.3390/genes17060664 (PMC13299357; doi:10.3390/genes17060664)
Supplement: Supplementary file 1 [file genes-17-00664-s001.zip › genes-4356227-supplementary.pdf]

Pyroptosis–ferroptosis crosstalk suggests candidate molecular clusters and immune remodeling in peri-implantitis

Author list:

Xinda Li[1,2#], Zhijia Liu[2#], Jiaxuan Nie[3], Jianing Wang[1,2], Jinlai Bao[4], Wuwei Li[1,2*]

Affiliations

1. Department of Oral and Maxillofacial Surgery, School of Stomatology, Dalian Medical University, Dalian, Liaoning, China

2.School of Stomatology, Dalian Medical University, Dalian, Liaoning, China

3.School of Stomatology, Changsha Medical University, Changsha, Hunan, China

4.School of Stomatology, Dalian University, Dalian, Liaoning, China

# These authors contributed equally to this work.

Corresponding Author
Wuwei Li, DDS.

Department of Oral and Maxillofacial Surgery,

School of Stomatology, Dalian Medical University,

Liaoning, China 116044

E-mail: dlyklww96@163.com

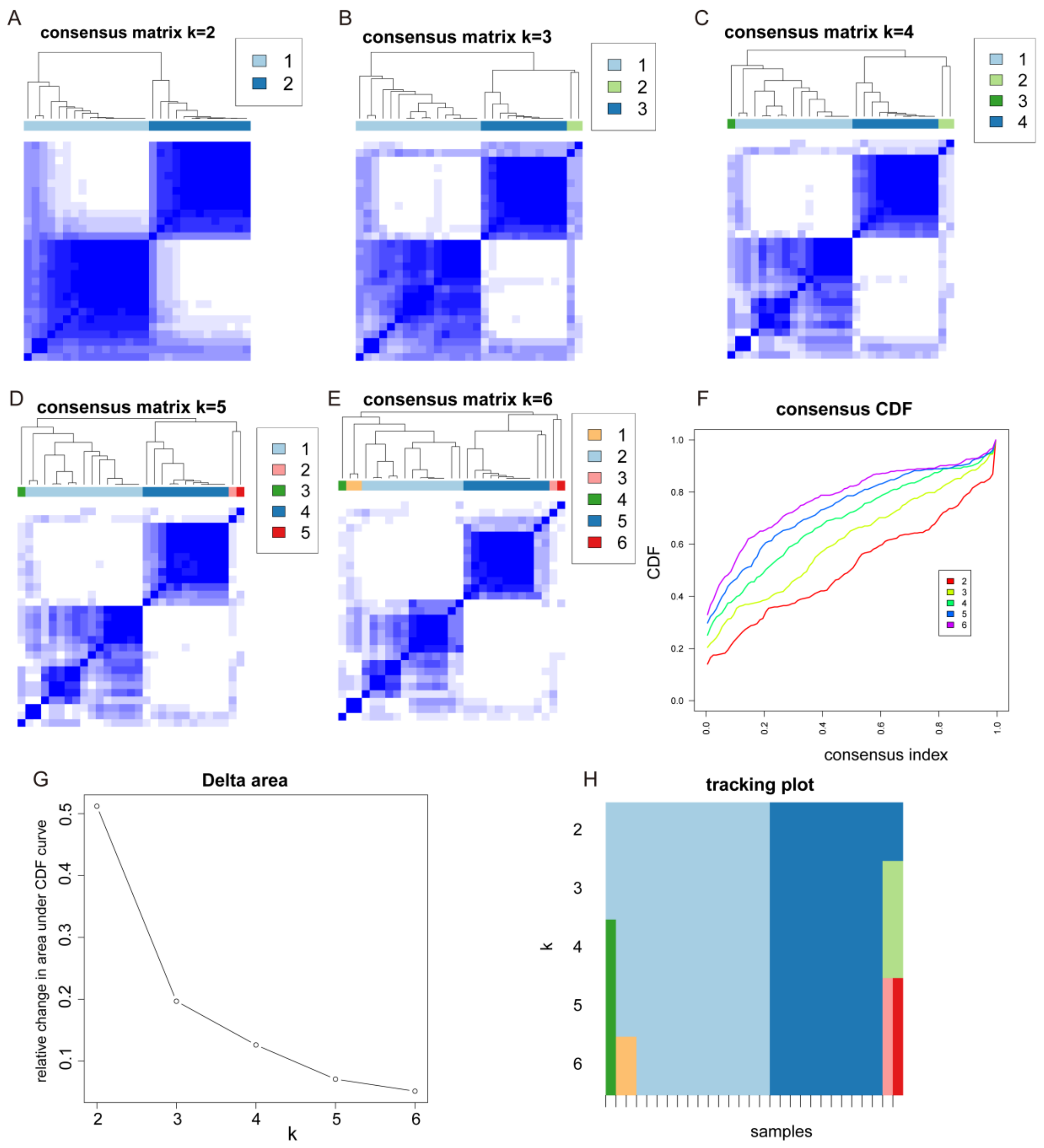

Figure S1. Consensus clustering evaluation based on the 41 pyroptosis–ferroptosis overlap genes.

(A–E) Consensus matrices for K = 2 to K = 6. (F) Cumulative distribution function (CDF) curves for different cluster numbers. (G) Delta area plot showing the relative change in the area under the CDF curve. (H) Tracking plot showing sample-cluster assignments across K = 2 to K = 6. Based on the consensus matrix, CDF curve, delta area plot, and tracking plot, K = 2 was selected for subsequent exploratory clustering analysis. The resulting C1/C2 groups were interpreted as candidate expression clusters rather than established molecular subtypes.

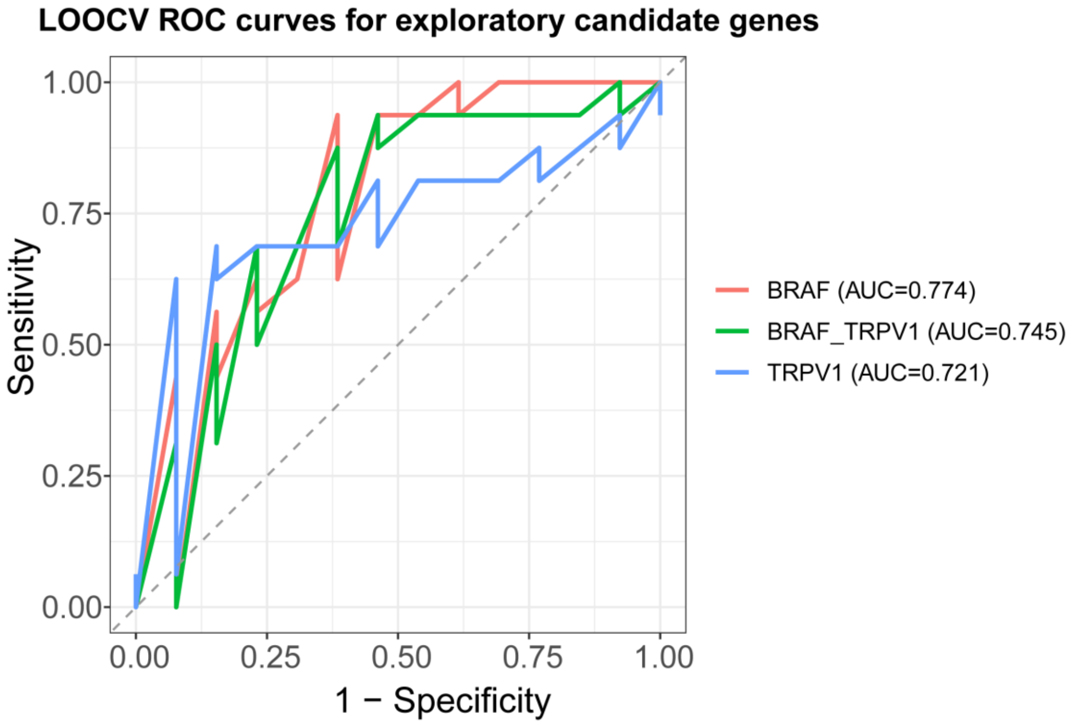

Figure S2. LOOCV ROC curves for exploratory candidate features.
Receiver operating characteristic curves were generated using leave-one-out cross-validation to evaluate the internal discriminatory performance of BRAF, TRPV1, and the combined BRAF + TRPV1 logistic model in the integrated cohort. The LOOCV AUC values were 0.774 for BRAF, 0.721 for TRPV1, and 0.745 for the combined model. These results represent exploratory internal performance rather than validated diagnostic utility.

Supplementary Table S1. Sources of pyroptosis-related genes

Source	Raw gene count
GeneCards	681
GSEA	27
Literature-curated list	101
Total entries	809
Unique genes after harmonization	741

Supplementary Table S2. Final non-redundant pyroptosis-related gene set

Gene symbol
ZBP1
TRPM7
TRAF2
TP63
TP53
TNFSF10

TNFAIP3
TNF
TLR4
TLR3
STAT5A
STAT3
STAT2
STAT1
SQSTM1
RIPK3
RIPK1
PYCARD
PGAM5
PARP1
NLRP3
MLKL
JAK2
IRF9
IRF2
IRF1
IL33
IL1B
IL1A
IL18
IFNG
HMGB1
GZMB
GSDME
GSDMD
FTH1
FADD
ELANE
EIF2AK2
DNM1L
CYLD
CHMP7
CHMP6
CHMP4C
CHMP4B
CHMP4A
CHMP3
CHMP2B
CHMP2A
CASP8

CASP5
CASP4
CASP3
CASP1
CAPN2
CAPN1
BIRC3
BCL2
BAX
BAK1
ALOX15
ZNF532
ZNF329
ZFAS1
ZDHHC9
ZDHHC7
ZDHHC5
ZDHHC1
YTHDF2
YAP1
YAF2
XIAP
XBP1
WTAP
WEE1
VSIG4
VPS4A
VPS4
VPS24
VHL
VEGFA
VDR
VDAC3
VDAC2
VDAC1
UTS2
USP7
USP48
USP25
USP21
USP18
USP14
USP1
USF2

ULK1
UHRF1
UCP1
UBR2
TYK2
TXNIP
TUG1
TSLP
TRPV1
TRIM6
TRIM59
TRIM45
TRIM38
TRIM31
TRIM29
TRIM25
TRIM24
TRIM21
TRIF
TRE-TTC3-1
TREM2
TREM1
TRA-TGC7-1
TRAF6
TRAF5
TRAF3
TRADD
TPM3
TP53TG1
TOMM20
TNFSF6
TNFSF15
TNFSF11
TNFRSF6
TNFRSF21
TNFRSF1A
TNFRSF11B
TNFRSF10B
TNFRSF10A
TNFAIP8L2
TLR9
TLR2
TIRP
TIGAR

TIFAB
TIFA
TFEB
TFAM
TEX14
TET2
TET1
TBK1
SYVN1
SYK
STK4
STING1
STAT6
STAT5B
STAT4
SSR1
SRP14
SREBF1
SPTBN1
SPTAN1
SPATA2
SOD2-OT1
SOCS3
SOCS1
SNRPN
SNORD15A
SNIP1
SNHG7
SNHG12
SNHG1
SNAP25
SMURF2
SMPD1
SMC4
SMARCA4
SMAD7
SMAD3
SMAD2
SLTM
SLC7A11
SLC6A14
SLC5A2
SLC4A2
SLC46A2

SLC39A5
SLC30A7
SLC2A1
SLC25A4S
SLC16A4
SIRT6
SIRT3
SIRT2
SIRT1
SIGLEC15
SHC1
SHARPIN
SGPP1
SGK1
SFTA1P
SFN
SF3B2
SF3B1
SF3A2
SETD7
SESN2
SERPINH1
SERPINC1
SERPINB1
SENP3
SDHB
SCUBE3
SCGB1A1
SCARNA5
SCAF8
SAMHD1
S1PR2
S100A9
S100A8
S100A4
S100A12
RSPO3
RSL1D1
RSF1
RRBP1
RPTOR
RPS19
RPL7A
RPL3

RPL27A
ROCK1
RNF31
RELA
RCN1
RBMX
RBM26
RBCK1
RBBP4
RASGRF1
PYG
PWARSN
PWAR1
PVT1
PVALB
PTX3
PTPN11
PTGS2
PTEN
PTBP1
PSMD2
PRORP
PROM2
PRMT5
PRG2
PRF1
PRDX1
PRDM1
PPM1D
PPID
PPIC
PPIA
PPARGC1A
PPARG
PLK4
PLAUR
PLA2R1
PLA2G4
PKM
PJVK
PJA1
PINK1
PIF1
PIEZO1

PICART1
PGR-AS1
PGM5-AS1
PGF
PFKFB3
PELP1
PELI2
PELI1
PDIA3
PDCD1
PCSK9
PANX1
PAK2
PAH
P4HA1
P2RX7
OTUD4
OPN3
OIP5-AS1
OGT
OGA
NSD2
NR4A1
NQO1
NPM1
NOX4
NOX2
NORAD
NOP56
NOP2
NOD2
NLRX1
NLRP9
NLRP7
NLRP6
NLRP2
NLRP1
NLRC4
NLRC3
NINJ1
NFKBIA
NFKB1
NFE2L2
NEXN

NEK7
NEDD4
NEAT1
NDUFA13
NDRG2
NCOA4
NAIP
MYH9
MYD88
MVP
MUC1
MTOR
MRE11
MPEG1
MOK
MMP9
MIR96
MIR9-2HG
MIR7-3HG
MIR582
MIR556
MIR543
MIR532
MIR527
MIR520C
MIR494
MIR485
MIR449A
MIR448
MIR433
MIR425
MIR424
MIR379
MIR34C
MIR342
MIR320D2
MIR320D1
MIR320C2
MIR320C1
MIR320B2
MIR320B1
MIR320A
MIR3163
MIR30E

MIR30C1
MIR302C
MIR302A
MIR26A2
MIR26A1
MIR23A
MIR223
MIR22
MIR216A
MIR215
MIR214
MIR21
MIR20B
MIR200C
MIR200B
MIR200A
MIR195
MIR193A
MIR18A
MIR185
MIR182
MIR17
MIR155HG
MIR155
MIR152
MIR141
MIR138-2
MIR138-1
MIR137
MIR135B
MIR1290
MIR125A
MIR124-1
MIR106B
MIR106A
MIR100HG
MINK1
MIAT
MFHAS1
METTL3
METTL14
MELTF
MELK
MEG3

MEFV
MDM2
MDH1
MCL1
MARCHF7
MAPKAP1
MAPK3
MAPK14
MAPK11
MAPK1
MAP3K5
MAP3K20
MAP2K6
MAP2K3
MAP1LC3B
MAP1LC3A
MALT1
MALAT1
LMNA
LINC02605
LINC02604
LINC02446
LINC01871
LINC01315
LINC01224
LINC01133
LINC01128
LINC00926
LINC00665
LINC00402
LINC00320
LINC00265
LINC00240
LINC00205
LIN28B
LETMD1
LDLR
LDHA
LCN2
KXD1-AS1
KRT6A
KRT14
KLK2
KLK1

KLF9
KLF6
KLF4
KLF3-AS1
KLF2
KIF23
KIF15
KIF11
KEAP1
KDM6B
KDM2A
KCNQ1OT1
KCNK5
JNK
JAK3
JAK1
ITPR2
ITGB5
ITGB2-AS1
IRF3
IRAK4
IQGAP1
INPP5D
IL6
IL37
IL32
IL17A
IL10
IKZF1
IKBKG
IKBKE
IGF2BP2
IGF2-AS
IGF2
IGF1
IFNGR2
IFNGR1
IFNB
IFNAR2
IFNAR1
IFNA
IFIT3
IFIT1
IFI27

IFI16
HULC
HTRA1
HSPA5
HSP90AB1
HSP90AA1
HSP90A
HP
HOTTIP
HOTAIRM1
HOTAIR
HMOX1
HMGCR
HMGB3
HIF1A
HIC1
HEATR5A
HDAC6
HDAC4
HDAC2
HDAC1
HCG18
HAGLR
H4C8
H4C4
H4C3
H4C2
H4C16
H4C15
H4C14
H4C13
H4C12
H4C11
H4C1
H3C13
H3C12
H3-3B
H2BC21
H2AZ2
H2AX
H2AC6
H2AC20
H2A
H19

H1-5
H1-2
H1-1
GZMA
GTF2H1
GSTP1
GSDMC
GSDMB
GSDMA
GRK2
GPX3
GLUD2
GLUD1
GLNA
GJB1
GJA1
GGT1
GBP1
GATA6
GATA1
GAS5
GAR1
GALNS
GABRG3
GABPB1-AS1
GABARAPL2
GABARAPL1
GABARAP
FZD6
FYN
FUS
FUNDC1
FTO
FTL
FSTL1
FSCN1
FPR2
FOXP3
FOXP1
FOXO4
FOXO3
FOXO1
FOXC1
FOXA2

FNDC5
FNDC4
FMR1
FLNA
FKBP10
FGFR2
FGF5
FGF23
FGF21
FFAR4
FCGRT
FAT1
FASN
FANCM
FAM3A
FAF1
EZH2
ETS1
ESR1
ERN1
ERAP2
EPHA2
EPB41L4A-AS1
EP300
ENTPD1-AS1
ELAVL1
EIF4A3
EIF2AK3
EGFR
EEF2K
E2F1
DUSP4
DUSP2
DUOX1
DTX3L
DRD2
DPP9
DPP8
DPP4
DNMT3A
DNMT1
DLX6-AS1
DLX6
DHX9

DHX8
DENND4C
DDX5
DDX3X
DDIT3
DCDC2
CYTOR
CYP1A1
CYCS
CYBB
CXCR4
CXCL8
CXCL12
CUL4B
CTSV
CTSG
CTSB
CTNNB1
CRTAC1
CRMA
CRLF1
CRIM1-DT
CPTP
CPA3
COL2A1
COL1A2
CMA1
CLIC4
CLEC7A
CLEC5A
CLEC3B
CITED2
CIB2
CIB1
CHRFAM7A
CHMP5
CHMP1
CGAS
CFLAR
CERNA3
CEBPB
CDKN1B
CDK9
CDCA3

CDC5L
CD55
CD274
CCR2
CCND2-AS1
CCND2
CCND1
CCL2
CCL15-CCL14
CBL
CASP7
CASP6
CARD8
CAMP
CAMK2
CALM3
CALM2
CALM1
CA9
CA1
BTN3A1
BSG
BRD4
BRCC3
BRCA1
BRAF
BNIP3
BMPR2
BIRC2
BID
BDNF-AS
BCL6
BBC3
AZU1
AZGP1
ATP6AP1
ATP2B1
ATG5
ASIC1
ARID5B
ARID1A
AREG
AQP1
APOL1

APOE
APOC3
APOA1
APIP
ANXA3
ANXA2
ANXA1
ANLN
ANKLE2
AMIGO2
ALOX5
ALKBH5
ALDH2
ALDH1L1-AS2
AKT1
AIM2
AIFM1
AHR
AGER
ADIPOQ
ADAR
ADAMTS9-AS2
ACTR2
ACTN4
ACTG1
ACTA2
ACSM3
ACE2
ABL1
ABCA1
MIR548X

This table contains the final harmonized pyroptosis gene set used for downstream analyses.

Supplementary Table S3. Sources of ferroptosis-related genes

Source	Raw gene count
GeneCards	929
GSEA	64
KEGG	41
Literature-curated list	88
Total entries	1122
Unique genes after harmonization	941

Supplementary Table S4. Final non-redundant ferroptosis-related gene set

Gene symbol
ZEB1
VDAC3
VDAC2
TXNRD1
TP53
TFRC
TF
STEAP3
SQLE
SLC7A11
SLC40A1
SLC3A2
SLC39A8
SLC39A14
SLC38A1
SLC1A5
SLC11A2
SAT2
SAT1
PTGS2
PROM2
PRNP
POR
PHKG2
PEBP1
PCBP2
PCBP1
OTUB1
NQO1
NOX4
NOX1
NFS1
NFE2L2
NCOA4
MT1G
MAP1LC3C
MAP1LC3B
MAP1LC3A
LPCAT3
KEAP1
IREB2
HSPB1
HSBP1

HMOX1
HMGCR
GSS
GPX4
GOT1
GLS2
GCLM
GCLC
GCH1
FTMT
FTL
FTH1
FDFT1
FANCD2
FADS2
DPP4
CYBB
CTH
CRYAB
CP
COQ2
CISD1
CHMP6
CHMP5
CHAC1
CD44
CBS
BACH1
ATG7
ATG5
ALOX5
ALOX15
ALOX12
AKR1C3
AKR1C2
AKR1C1
AIFM2
ACSL6
ACSL5
ACSL4
ACSL3
ACSL1
ACO1
ZNF8

ZNF667-AS1
ZNF350
ZFP36
ZFAS1
ZEB1-AS1
ZDHHC8
ZDHHC16
ZC3H13
ZBTB48
ZBTB20
YY2
YY1AP1
YY1
YWHAE
YTHDF2
YTHDF1
YTHDC2
YTHDC1
YBX1
YAP1
XRCC6
XPO1
WWTR1
WWOX
WTAP
WEE1
WDR76
WDR5
VIRMA
VIM
VEGFA
VDAC1
VCP
VAMP8
UTP11
USP8
USP7
USP5
USP48
USP35
USP3
USP22
USP2
USP19

USP18
USP14
USP11
USF2
USF1
URB1
UHRF1
UFL1
UCHL5
UCHL3
UBR5
UBE2N
UBE2K
TYRO3
TYMS
TXNIP
TWIST1
TUG1
TTBK2
TRU-TCA1-1
TRPV4
TRPV1
TRPM7
TRPM4
TRPA1
TRIM7
TRIM69
TRIM59
TRIM54
TRIM37
TRIM36
TRIM33
TRIM3
TRIM28
TRIM26
TRIM22
TRIM21
TRIM11
TRIB3
TRC-GCA24-1
TRA-TGC7-1
TPM4
TPM3
TP63

TOP2A
TOMM20
TNFSF9
TNFSF4
TNFSF11
TNFSF10
TNFAIP3
TNF
TMPO-AS1
TMEM164
TLR4
TLR3
TLR2
TIMP1
TIGAR
TIFAB
TGFBR1
TGFB1
TFR2
TFEB
TFE3
TFCP2L1
TFAP2C
TFAP2A
TFAM
TET2
TEAD4
TEAD1
TCL6
TCF21
TBRG4
TBK1
TBC1D5
TAX1BP1
SYVN1
SYNPR-AS1
SUMO1
STYK1
STUB1
STK11
STING1
STEAP1
STC2
STAT6

STAT3
STAT1
SSBP1
SRXN1
SRSF3
SRSF2
SRSF1
SREBF2
SREBF1
SQSTM1
SPTBN2
SPOP
SPINK2
SPC25
SPARC
SP1
SOX9
SOX4
SOX2
SOX15
SOD2-OT1
SOD1
SOCS2
SOCS1
SNRPB
SNORD15A
SNHG7
SNHG4
SNHG17
SNHG16
SNHG14
SNHG10
SNHG1
SND1
SNCA
SNAI2
SNAI1
SMG9
SMARCB1
SMARCA4
SMAD3
SMAD2
SLCO4A1-AS1
SLC7A5

SLC6A14
SLC47A1
SLC39A7
SLC38A5
SLC35F2
SLC2A3
SLC2A12
SLC2A1
SLC27A5
SLC27A4
SLC25A28
SLC25A10
SLC25A1
SLC12A5
SLAMF8
SKP2
SIRT6
SIRT3
SIRT2
SIRT1
SHH
SHC1
SHARPIN
SFTA1P
SF3B2
SF3B1
SETDB1
SETD7
SESN2
SENP3
SENP1
SELENOP
SELENBP1
SEC24B
SCUBE3
SCD
SCARNA5
SCARNA22
SCARA5
SC5D
SAFB2
S100A4
RUVBL1
RUNX3

RUNX2
RPS6
RPS3A
RPL8
RPL7
RPL5
RPL13A
ROCK2
ROCK1
RMST
RIPK3
RIPK1
RHOT1
RETREG1
RELA
RBMX
RBMS1
RBM4
RBM15
RBCK1
RB1CC1
RB1
RACK1
RAB14
QSOX1
PVT1
PTPN6
PTK2
PTGER3
PTEN
PTBP1
PSMA3-AS1
PSEN1
PRRX2
PRMT6
PRMT5
PRMT1
PRKDC
PRKAA2
PRKAA1
PRDX6
PRDX5
PRDX2
PRDX1

PPT1
PPP2CA
PPARGC1A
PPARG
PPARA
POU6F1
POU2F2
PORCN
POLE2
PNO1
PML
PLXNB2
PLTP
PLIN2
PLAG1
PLA2G6
PLA2G4A
PKN2
PKM
PITX2
PIR
PINK1
PIM1
PIEZO1
PIAS1
PHB2
PHB1
PGRMC1
PGR-AS1
PGK1
PGD
PGAM5
PFKFB3
PELATON
PEDS1
PDK4
PDIA6
PDIA4
PDGFRB
PDE4B
PDCD1
PCSK9
PCNA
PCK2

PCDH7
PCBP3
PAX8
PARP1
PARL
PARK7
PAQR3
PANX1
PADI4
P4HA1
OTUD6B-AS1
OTUD5
OPA3
OIP5-AS1
OGT
OGFRP1
NUPR1
NSUN5
NSUN2
NRF2
NRAS
NR5A2
NR4A1
NR1H4
NPM1
NOTCH3
NOTCH2
NOTCH1
NKAP
NINJ1
NGLY1
NFKB1
NFE2L1
NF2
NEK2
NEDD4L
NEDD4
NEAT1
NDFIP1
NCK1-DT
NAT10
NABP2
MYO18A
MYLK-AS1

MYL6B
MYH9
MYEOV
MYCN
MYC
MYB
MUC1
MTOR
MTDH
MTCH1
MPHOSPH10
MPC1
MNT
MMP2
MMP13
MMD
MLKL
MKRN3
MITF
MITD1
MIRLET7C
MIR93
MIR9-1
MIR758
MIR7-3HG
MIR6852
MIR655
MIR545
MIR522
MIR507
MIR497
MIR494
MIR4668
MIR4465
MIR4443
MIR429
MIR425
MIR424
MIR375
MIR373
MIR372
MIR34A
MIR338
MIR326

MIR320A
MIR30E
MIR27A
MIR23A
MIR214
MIR193A
MIR188
MIR181D
MIR155HG
MIR142
MIR141
MIR133B
MIR129-1
MIR1287
MIR122
MIR100HG
MIOX
MICU1
MIB2
MIAT
MGST1
MFGE8
MEX3A
METTL3
METTL17
METTL16
METTL14
MERTK
MEN1
MELTF
MELK
MEF2C
MEF2A
MDM4
MDM2
MCOLN1
MCM4
MBOAT7
MAT2A
MARCHF6
MAPT-AS1
MAPK3
MAP4
MAP3K5

MAP1LC3B2
MAL2
MAFG
LUCAT1
LTF
LRPPRC
LPIN1
LPCAT1
LONP1
LMNB1
LIPE-AS1
LINC03122
LINC02605
LINC02446
LINC01871
LINC01857
LINC01833
LINC01770
LINC01614
LINC01572
LINC01503
LINC01224
LINC01133
LINC01063
LINC00968
LINC00665
LINC00472
LINC00462
LINC00336
LINC00324
LINC00205
LILRB1
LIFR
LGI1
LGALS3
LGALS1
LDHB
LDHA
LCN2
LATS2
LASTR
LASP1
LAMP2
LACTB

KTN1-AS1
KRT6B
KRT19
KRT18
KRT16
KRT14
KRAS
KMT2A
KLK2
KLF5
KLF4
KLF2
KLF15
KLF11
KLB
KL
KDM6B
KDM5C
KDM5A
KDM4D
KDM4A
KDM3B
KDM1A
KCTD10
KAT5
KAT2B
KAT2A
JUN
JMJD6
JAM3
JAK2
JAK1
ITGB8
ITGB1
ITGA6
ITCH
ISCU
ISCA2
IRF1
ILF3
IL6
IL4I1
IL37
IL1B

IKBKG
IGKC
IGHG4
IGF2BP3
IGF2BP2
IGF2BP1
IGF1R
IFNG
IFITM9P
IDO1
IDH2
IDH1
IARS1
HULC
HSPA9
HSPA8
HSPA5
HSP90B1
HSDL2
HSD17B11
HOTAIRM1
HNRNPM
HNRNPD
HNRNPC
HNRNPA3
HNRNPA2B1
HNRNPA1
HNF4A
HMGCS1
HMGB2
HMGB1
HIVEP3
HIPK3
HIF1A
HIC1
HERPUD1
HEPFAL
HELLS
HECW1
HECTD3
HDLBP
HDDC3
HDAC9
HDAC6

HDAC3
HDAC2
HDAC1
HCRT
HCG18
HCAR1
HAS2
HAMP
HACE1
H2AX
H2AC1
H19
H1-3
H1-2
GTF2H1
GSTZ1
GSTP1
GSTO2
GSTM3
GSK3B
GSDME
GSDMD
GRIK1-AS2
GPX3
GPX1
GPR68
GPD2
GOLPH3
GJA1
GDF15
GCN1
GATA3
GAS5
GALNT14
GADD45A
GABPB1-AS1
GABPA
GABARAPL1
G6PD
G3BP1
FZD7
FXR1
FXN
FUS

FUNDC1
FTO
FSTL1
FSCN1
FPR2
FOXP1
FOXO3
FOXM1
FOXC1
FOXA2
FOSL2
FNDC5
FMR1
FLVCR2
FLT3
FLRT2
FKBP3
FHOD1
FGFR2
FGFR1
FGF4
FGF21
FFAR2
FERRISR
FCN3
FBXW7
FBXO31
FBLN1
FASN
FAM120A
FABP4
FABP1
F11R
EZH2
ETV4
ETS1
ESR1
ERN1
ERBB2
EPHA2
EP300
ENO1
EMP1
EMC2

ELK4
ELF3
ELAVL1
EIF4EBP1
EIF2AK4
EIF2AK3
EHMT2
EGLN1
EGFR
EFEMP1
EEF1A1
ECH1
E2F1
DUSP4
DUOX1
DPP9
DPEP1
DNMT3B
DNMT1
DNM1L
DNAJB6
DLEU1
DKK1
DHX9
DHODH
DHCR7
DECR1
DDX3X
DDR2
DDR1
DDIT4
DDIT3
DDHD2
DAZAP1
CYTOR
CYP2J2
CYP24A1
CYP1B1
CYLD
CYGB
CYB5R1
CXCL12
CX3CL1
CUL4B

CTSB
CTNNB1
CST1
CS
CRY2
CRMA
CRIM1
CREBBP
CREB1
CPT1A
CPEB4
CPEB1
COX7A1
COPZ1
COMMD10
COL5A1
COL1A1
CNDP1
CMTM5
CKS2
CKB
CISD3
CISD2
CIRBP
CHMP1A
CGAS
CERS6-AS1
CERS6
CERNA3
CEPT1
CEP290
CELF2
CDO1
CDKN2A
CDK6
CDK14
CDK12
CDK1
CDH2
CDCA7
CDC25A
CD82
CD47
CD36

CD276
CD274
CCT3
CCND1
CCL5
CCDC144NL-AS1
CBSLR
CAVIN1
CAV1
CASP3
CARS1
CARS
CAMKK2
CALR
CA9
C1RL-AS1
C1QTNF6
C1QL3
C19orf12
BUB1B
BSG
BRD7
BRD4
BRCA1
BRAF
BNIP3L
BNIP3
BMAL1
BIRC5
BGN
BECN1
BDNF-AS
BCLAF1
BCL2
BBOX1-AS1
BAP1
B3GNT3
AXL
AURKB
AURKA
ATP5MC3
ATP5F1B
ATP5F1A
ATM

ATL1
ATG16L1
ATF6
ATF4
ATF3
ATF2
ASMTL-AS1
ASCL4
ASCL1
ASAH2
ARRDC1-AS1
ARPC1A
ARHGEF26-AS1
ARHGAP6
ARG2
AR
APP
APOL3
APOE
APOC1
APEX1
ANXA3
ANXA2
ANXA1
ANO1
ANKRD1
AMER1
AMBRA1
ALKBH5
ALG3
ALDH2
ALDH1A1
ALB
ALAS2
AKT1
AKR1B1
AKAP1
AIM2
AIFM1
AHR
AHCY
AFF3
ADIPOR1
ADGRG1

ADCY10
ADAR
ADAMTS9-AS1
ACTN4
ACTB
ACTA2
ACSF2
ACOT7
ACO2
ACLY
ACACA
ABCC5
ABCC2
ABCC1
ABCB6
ABCB10
ABCB1
A2M-AS1

This table contains the final harmonized ferroptosis gene set used for downstream analyses.

Table S5. Sensitivity analysis of differential-expression thresholds

Threshold	Total candidate genes	Upregulated genes	Downregulated genes
Adjusted P value < 0.30 and |log2FC| ≥ 0.15	2,450	1,280	1,170
Nominal P value < 0.05 and |log2FC| ≥ 0.15	2,617	1,351	1,266
Adjusted P value < 0.25 and |log2FC| ≥ 0.15	1,796	986	810
Adjusted P value < 0.05 and |log2FC| ≥ 0.15	12	12	0

The original exploratory screening threshold was adjusted P value < 0.30 and |log2FC| ≥ 0.15. More stringent thresholds were used as sensitivity analyses to evaluate the influence of DEG selection criteria. Genes identified under the original threshold were interpreted as exploratory candidate differentially expressed genes rather than definitive disease-driving genes.

Table S6. Complete GSEA pathway-level statistics.

Panel A. Enrichment statistics.

No.	Pathway	Set size	Enrichment score	NES	Nominal P value	Adjusted P value	Rank
1	ALLOGRAFT REJECTION	191	0.6623	2.7018	1.456e-25	7.280e-24	2149

No.	Pathway	Set size	Enrichment score	NES	Nominal P value	Adjusted P value	Rank
2	COMPLEMENT	194	0.643	2.6291	8.981e-23	2.245e-21	1851
3	INFLAMMATORY RESPONSE	197	0.6339	2.5951	6.257e-21	1.043e-19	2503
4	Epithelial Mesenchymal Transition	192	0.6046	2.4704	3.731e-18	4.664e-17	2246
5	INTERFERON GAMMA RESPONSE	192	0.6025	2.4622	6.507e-18	6.507e-17	2422
6	KRAS SIGNALING (Up)	190	0.6038	2.4671	2.669e-17	2.224e-16	2223
7	mTORC1 SIGNALING	189	0.5841	2.3872	8.407e-16	6.005e-15	3109
8	TNFα SIGNALING VIA NF-κB	194	0.5748	2.3503	4.533e-15	2.833e-14	2117
9	IL-2 STAT5 SIGNALING	191	0.5685	2.3192	1.288e-14	7.157e-14	2827
10	OXIDATIVE PHOSPHORYLATION	179	0.5577	2.2613	4.326e-13	2.163e-12	5209
11	COAGULATION	136	0.5716	2.2468	9.956e-11	4.526e-10	1802
12	IL-6 JAK STAT3 SIGNALING	83	0.6233	2.3138	5.828e-09	2.428e-08	2503
13	APOPTOSIS	156	0.5279	2.1161	6.329e-09	2.434e-08	2620
14	MYC TARGETS V1	188	0.4674	1.9088	2.957e-07	1.056e-06	5045
15	UNFOLDED PROTEIN RESPONSE	105	0.5341	2.0361	3.115e-06	1.038e-05	2900
16	UV RESPONSE (Up)	152	0.4621	1.8436	2.052e-05	6.412e-05	2958
17	ADIPOGENESIS	188	0.427	1.7437	2.782e-05	8.078e-05	4229

No.	Pathway	Set size	Enrichment score	NES	Nominal P value	Adjusted P value	Rank
18	KRAS SIGNALING (Down)	188	-0.3505	-1.6742	2.908e-05	8.078e-05	4780
19	REACTIVE OXYGEN SPECIES PATHWAY	46	0.592	1.9562	1.235e-04	3.251e-04	2620
20	p53 PATHWAY	188	0.4027	1.6446	2.637e-04	6.594e-04	4097
21	ANDROGEN RESPONSE	96	0.4698	1.7706	3.258e-04	7.758e-04	2327
22	PROTEIN SECRETION	93	0.4729	1.7746	5.058e-04	0.0011	4457
23	ANGIOGENESIS	36	0.6035	1.907	5.626e-04	0.0012	2311
24	GLYCOLYSIS	195	0.3931	1.6091	7.341e-04	0.0015	3729
25	E2F TARGETS	192	0.3845	1.5712	8.369e-04	0.0017	3951
26	XENOBIOTIC METABOLISM	195	0.3605	1.4758	0.0062	0.012	1921
27	G2/M CHECKPOINT	187	0.3475	1.4174	0.0125	0.0231	3854
28	CHOLESTEROL HOMEOSTASIS	70	0.4371	1.5664	0.0176	0.0315	2327
29	HYPOXIA	189	0.3376	1.3797	0.0227	0.038	3752
30	APICAL JUNCTION	191	0.3353	1.3679	0.0228	0.038	3079
31	MITOTIC SPINDLE	196	0.3407	1.3957	0.0255	0.0412	4619
32	TGF-β β SIGNALING	53	0.4433	1.5069	0.0277	0.0434	2982
33	INTERFERON ALPHA RESPONSE	91	0.3842	1.439	0.0352	0.0534	3278
34	PEROXISOME	102	0.3648	1.3897	0.0373	0.0548	2374
35	FATTY ACID METABOLISM	152	0.3446	1.3748	0.0406	0.058	2416

No.	Pathway	Set size	Enrichment score	NES	Nominal P value	Adjusted P value	Rank
36	UV RESPONSE (Down)	135	0.3377	1.3247	0.0537	0.0746	2646
37	SPERMATOGE NESIS	132	-0.2817	-1.2858	0.0574	0.0757	2001
38	MYOGENESIS	196	-0.2614	-1.2482	0.0575	0.0757	3534
39	(Down)A REPAIR	137	0.3335	1.3117	0.065	0.0833	5259
40	PI3K AKT mTOR SIGNALING	103	0.3502	1.3325	0.0668	0.0836	3521
41	APICAL SURFACE	43	0.4096	1.3367	0.0893	0.1089	1505
42	Wnt β Catenin SIGNALING	42	-0.3662	-1.3612	0.1112	0.1324	3278
43	HEME METABOLISM	184	0.2772	1.1282	0.2094	0.2435	4557
44	BILE ACID METABOLISM	112	0.295	1.1354	0.2244	0.2494	1990
45	ESTROGEN RESPONSE EARLY	191	0.2744	1.1194	0.2222	0.2494	2108

Panel B. Leading-edge summary and core enrichment genes.

No.	Pathway	Leading-edge summary	Core enrichment genes
1	ALLOGRAFT REJECTION	tags=45%, list=13%, signal=40%	CD79A, IL1B, SRGN, CXCL13, IFNAR2, CTSS, PTPRC, IRF4, IGSF6, CCR1, GPR65, FCGR2B, LYN, ST8SIA4, IL6, NCF4, HLA-DOB, HLA-DRA, TIMP1, ETS1, TLR1, LCP2, TGFB1, TLR6, CD80, IL2RG, IFNGR1, BCAT1, CD3G, B2M, IL18RAP, CD74, HCLS1, ITGB2, IL2RA, RIPK2, F2R, HLA-DQA1, ITGAL, MMP9, HLA-DMA, GALNT1, CD86, THY1, CCL19, ICAM1, GZMA, NME1, TLR2, IRF8, CD4, STAT4, C2, CCL5, PRKCB, IL10, HLA-DMB, UBE2D1, CD2, NLRP3, CD3E, CD28, IL2RB, SOCS1, CCL22, CD40LG, CD3D, TRAT1, CD96, FAS, CCL2, MAP4K1, ABCE1, GBP2, IL27RA, HIF1A, IL18, CCR2, INHBA, CDKN2A, BCL3, NPM1, DYRK3, BCL10, LY86, BRCA1
2	COMPLEMENT	tags=40%, list=11%, signal=36%	MMP12, FCER1G, C3, CTSS, GCA, CXCL1, GZMK, CTSH, PLAT, MMP13, LYN, DUSP6, PLEK, PLA2G7, S100A12, IL6, PIK3CG, CASP10, CD46, C1QC, APOBEC3G, OLR1, CD59, KYNU, TIMP1,

No.	Pathway	Leading-edge summary	Core enrichment genes
			LCP2, CFH, SERPINA1, CTSB, PLAUR, LTA4H, CTSC, HSPA5, TNFAIP3, PLSCR1, PRCP, CASP5, FN1, ANXA5, DUSP5, C1QA, COL4A2, GZMA, CSRP1, ADAM9, CASP4, CR1, RASGRP1, ITGAM, C2, USP15, CASP1, C1S, ZEB1, APOC1, SERPINE1, CASP3, DOCK4, DOCK10, CA2, CCL5, CFB, LGMN, FYN, APOBEC3F, S100A9, CD40LG, CDA, C1R, PDP1, CR2, CLU, GRB2, SERPING1, PRKCD, LIPA, USP16
3	INFLAMMATORY RESPONSE	tags=46%, list=15%, signal=40%	IL1B, RGS1, AQP9, KCNA3, CD14, CYBB, IL1A, SELE, MSR1, TNFAIP6, C3AR1, IL10RA, LYN, FPR1, CSF3R, IL6, SELL, CD48, OLR1, TIMP1, CD69, TLR1, LCP2, CALCRL, CCL20, IL7R, PDPN, GPR183, CXCL6, GPC3, ITGA5, PLAUR, BTG2, AHR, HBEGF, SLAMF1, MXD1, APLNR, C5AR1, IL1R1, IL18RAP, CMKLR1, PROK2, RIPK2, FFAR2, ICAM1, CCRL2, TLR2, PTPRE, RASGRP1, IFNAR1, SPHK1, HAS2, SERPINE1, CCL5, IL10, EMP3, NLRP3, PDE4B, GPR132, IL2RB, CCL22, ATP2A2, GNA15, LAMP3, OSMR, OSM, NAMPT, CCL2, SGMS2, BEST1, CCR7, RGS16, HIF1A, IL18, PTAFR, INHBA, NMI, ADM, ATP2B1, PTGER4, IRF1, ITGB3, TNFRSF1B, SRI, IL4R, CHST2, NFKBIA, LPAR1, PIK3R5, IL18R1
4	Epithelial Mesenchymal Transition	tags=45%, list=14%, signal=40%	SFRP4, MMP1, MFAP5, MGP, CXCL1, SPP1, IL6, GLIPR1, POSTN, LUM, FSTL1, CALU, FAP, FBN1, CD59, TNC, NNMT, TIMP1, LAMC2, MMP3, CXCL6, TGFB1, PMP22, ITGA5, PLAUR, TAGLN, DAB2, TGM2, WIPF1, TNFAIP3, PMEPA1, COL4A1, ECM2, PTHLH, FN1, ADAM12, MXRA5, PLOD2, COL6A3, DPYSL3, NID2, ACTA2, THY1, COL4A2, GREM1, NT5E, FERMT2, GPX7, SERPINE1, LAMC1, EMP3, CALD1, THBS1, FBLN5, MMP2, CTHRC1, BGN, PLOD3, FUCA1, VCAM1, THBS2, MATN3, TIMP3, SERPINH1, TNFRSF12A, FMOD, TNFRSF11B, LAMA2, LGALS1, PDGFRB, FBLN1, FAS, PFN2, ECM1, TPM1, BMP1, SAT1, INHBA, ANPEP, LRRC15, CDH11, SDC4, ENO2, ITGB3, SERPINE2, MEST, ITGAV
5	INTERFERON GAMMA RESPONSE	tags=42%, list=15%, signal=36%	SLAMF7, CD38, IFNAR2, IRF4, PTGS2, SAMHD1, TNFAIP6, IL10RA, FPR1, SELP, ST8SIA4, IL6, FGL2, SOD2, CD69, LCP2, CFH, ZBP1, UPP1, IDO1, ST3GAL5, HLA-DRB1, TNFAIP3, SOCS3, B2M, CMKLR1, CD74, PNP, PLSCR1, RIPK2, SAMD9L, HLA-DQA1, HLA-DMA, CD86, ICAM1, GZMA, CASP4, IRF8, STAT4, CASP1, SPPL2A, BANK1, C1S, CASP3, CSF2RB, CCL5, CFB, PSMA3, ITGB7, PFKP, TDRD7, PDE4B, VCAM1, GBP4, IL2RB, SOCS1, IFITM2, BPGM, HLA-B, CD274, C1R, NAMPT, FAS, CCL2, MT2A, APOL6, SERPING1, HIF1A, NMI, MTHFD2, CASP8, IRF1, TNFAIP2, IRF5, PTPN2, SRI, IL4R, ISG20, IFITM3, NFKBIA
6	KRAS SIGNALING (Up)	tags=41%, list=13%, signal=36%	IL1B, ST6GAL1, LY96, FCER1G, CTSS, CXCR4, PTGS2, SPP1, PECAM1, PLAT, C3AR1, IL10RA, LAPTM5, DUSP6, IKZF1, LCP1,

No.	Pathway	Leading-edge summary	Core enrichment genes
			SLPI, TRIB1, DOCK2, PRDM1, TFPI, F13A1, ETS1, ADAMDEC1, CFH, CCL20, TMEM176A, IL7R, GNG11, PLAUR, HBEGF, SPARCL1, IL2RG, TSPAN1, TLR8, MMD, IL33, GLRX, TNFAIP3, CMKLR1, ITGB2, EPB41L3, EMP1, NRP1, GYPC, MMP9, CLEC4A, PDCD1LG2, IRF8, ALDH1A3, ENG, TOR1AIP2, PLAU, TSPAN13, CA2, CFB, TMEM176B, TSPAN7, MMP10, G0S2, CD37, FUCA1, BIRC3, ETV5, CBL, BPGM, GFPT2, SERPINA3, MAP4K1, RGS16, INHBA, ETV4, PPBP, FBXO4, SDCCAG8, TNFRSF1B, GPNMB, GALNT3
7	mTORC1 SIGNALING	tags=50%, list=19%, signal=41%	XBP1, CXCR4, SLC2A3, SKAP2, PDK1, FGL2, CORO1A, SLA, SLC7A11, HSP90B1, BTG2, NFIL3, LTA4H, CTSC, HSPA5, GLRX, BCAT1, CTH, PNP, ITGB2, TES, PLOD2, EDEM1, DDIT4, EEF1E1, DAPP1, ELOVL5, TFRC, RDH11, RPN1, SLC7A5, FADS1, CCNG1, PLK1, USO1, RAB1A, LGMN, PSMA4, STIP1, SLC1A5, PSMA3, SHMT2, YKT6, P4HA1, IDH1, PIK3R3, GOT1, ATP2A2, SSR1, UCHL5, SERPINH1, STARD4, FADS2, MTHFD2L, M6PR, TBK1, NAMPT, HSPE1, HPRT1, EIF2S2, LDHA, UBE2D3, TXNRD1, SDF2L1, IFRD1, MTHFD2, SLC1A4, ADD3, IMMT, SCD, PNO1, ATP6V1D, HSPA4, AURKA, STC1, IDI1, PRDX1, HSPD1, ASNS, ACLY, DDX39A, GSR, GLA, PSPH, CALR, RIT1, PSMD14, PSMD13, ARPC5L, SYTL2, CANX, BUB1, FKBP2, NUP205
8	TNFα SIGNALING VIA NF-κB	tags=35%, list=13%, signal=31%	IL1B, IL1A, CXCL1, PTGS2, TNFAIP6, SLC2A3, PLEK, IL6, BCL2A1, TRIB1, IL6ST, OLR1, SOD2, TNC, KYNU, CD69, CCL20, IL7R, GPR183, CXCL6, DRAM1, PLAUR, BTG2, HBEGF, MSC, CD80, MXD1, DNAJB4, NFIL3, TNFAIP3, PMEPA1, SOCS3, B4GALT1, RIPK2, MCL1, PHLDA1, DUSP5, ICAM1, PDLIM5, CCRL2, TLR2, PTPRE, SPHK1, SERPINE1, PLAU, CCL5, CXCL2, G0S2, PDE4B, LITAF, BIRC3, CFLAR, GFPT2, IER3, ZFP36, NAMPT, CCL2, DENND5A, SAT1, B4GALT5, IL18, INHBA, BCL3, ATP2B1, PTGER4, IRF1, SDC4, TNFAIP2
9	IL-2 STAT5 SIGNALING	tags=41%, list=17%, signal=35%	IRF4, XBP1, RHOH, SPP1, GPR65, SLC2A3, IL10RA, SELP, SELL, F2RL2, CD48, FGL2, PLIN2, ICOS, AHR, ODC1, MXD1, SLC39A8, IFNGR1, NFIL3, GLIPR2, TGM2, CTLA4, PNP, PLSCR1, LRRC8C, IL2RA, PHLDA1, EMP1, NRP1, CD86, MUC1, SYT11, IRF8, NT5E, ADAM19, CASP3, CA2, ITGA6, CDC42SE2, IL10, SLC1A5, ST3GAL4, P4HA1, BCL2L1, SPRY4, MAPKAPK2, GBP4, IL2RB, SOCS1, IL1RL1, CDC6, CTSZ, ENPP1, DENND5A, PTRH2, ECM1, RGS16, ALCAM, ETV4, IL1R2, TWSG1, PRNP, PHTF2, TNFRSF1B, ITGAV, IL4R, GALM, IFITM3, IL18R1, DCPS, PLAGL1, P2RX4, NCOA3, TNFRSF9, SERPINB6, SNX14, SHE, NFKBIZ
10	OXIDATIVE PHOSPHORYLATIO	tags=64%, list=32%, signal=44%	TIMM17A, COX7C, COX7B, ATP1B1, COX6C, MRPS15, UQCRQ, LDHB, SDHB, NDUFAB1, NDUFA2, COX7A2, SLC25A20, PDHA1,

No.	Pathway	Leading-edge summary	Core enrichment genes
	N		CYCS, NDUFA9, NDUFA6, ACADM, NDUFB3, ALAS1, NDUFB5, HADHB, IDH1, MGST3, ETFA, COX6A1, NDUFV2, HADHA, NDUFA1, ATP6V1C1, ECH1, DLD, UQCRC2, PDP1, OPA1, CPT1A, PDK4, LRPPRC, TIMM9, LDHA, ATP6V0B, MRPS22, SUCLG1, NDUFB6, NDUFA8, NDUFA4, MRPL15, UQCRB, ACAT1, COX17, IMMT, MTRR, ATP6V1D, COX5B, ATP6V0E1, PRDX3, MDH2, CYC1, COX4I1, NDUFS7, SURF1, NDUFA5, NDUFS4, OXA1L, NDUFV1, GRPEL1, DLAT, COX6B1, AIFM1, UQCRFS1, NDUFB8, MTX2, COX8A, UQCR11, ATP6V1E1, GPX4, TIMM8B, TCIRG1, ABCB7, UQCR10, GPI, IDH2, TIMM10, DECR1, IDH3A, PHYH, MDH1, COX7A2L, ISCU, UQCRC1, ETFB, COX5A, SUCLA2, FDX1, GLUD1, ACO2, NNT, SLC25A11, MRPS11, SDHA, CYB5A, ATP6V1G1, SDHC, BDH2, HSPA9, GOT2, SLC25A3, COX10, TIMM13, NDUFA7, NDUFS1, POR, TIMM50, MTRF1, ETFDH
11	COAGULATION	tags=35%, list=11%, signal=32%	MMP1, C3, MMP7, CTSH, PECAM1, PLAT, DUSP6, PLEK, F2RL2, FBN1, OLR1, TIMP1, A2M, CFH, SERPINA1, CTSB, MMP3, ANXA1, CFI, LTA4H, FN1, PRSS23, MMP9, C1QA, CTSK, CSRP1, ADAM9, VWF, C2, C1S, APOC1, SERPINE1, PLAU, CFB, LGMN, CRIP2, CAPN2, THBS1, MMP10, MMP2, RAC1, FYN, TIMP3, C1R, ARF4, CLU, SERPING1, BMP1
12	IL-6 JAK STAT3 SIGNALING	tags=47%, list=15%, signal=40%	IL1B, CXCL13, CD38, CD14, CXCL1, CCR1, CSF3R, IL6, IL6ST, A2M, TGFB1, IL2RG, IL10RB, IFNGR1, PLA2G2A, IL1R1, SOCS3, ITGA4, IL2RA, TLR2, BAK1, IFNAR1, CSF2RB, HAX1, CBL, SOCS1, TNFRSF12A, OSMR, FAS, GRB2, IL1R2, IRF1, ITGB3, ACVRL1, TNFRSF1B, PTPN2, IL4R, PIK3R5, IL18R1
13	APOPTOSIS	tags=38%, list=16%, signal=32%	IL1B, CD38, CD14, IL1A, PLAT, IL6, LUM, SOD2, TIMP1, CD69, DNAJC3, ANXA1, BTG2, IFNGR1, SLC20A1, DAP, CTH, NEDD9, MCL1, F2R, BCL2L11, EMP1, CASP4, CASP1, HGF, CASP3, ROCK1, BCL2L1, MMP2, BGN, CD2, BIRC3, BID, CFLAR, GNA15, TIMP3, TNFRSF12A, IER3, PDGFRB, FAS, CLU, SAT1, CYLD, DNAJA1, IL18, CASP8, WEE1, FEZ1, IRF1, BCL10, ENO2, BRCA1, ISG20, IFITM3, CDK2, PPT1, CAV1, AVPR1A, GSR
14	MYC TARGETS V1	tags=52%, list=31%, signal=36%	PRDX4, ODC1, KPNA2, PSMB3, TXNL4A, NME1, NDUFAB1, PTGES3, CCT5, VBP1, PSMA4, PSMA6, GNL3, SRSF3, NOLC1, PABPC4, PSMD8, BUB3, PSMD7, SNRPD1, NOP56, MAD2L1, EIF1AX, SSBP1, TYMS, ABCE1, HSPE1, HPRT1, EIF2S2, AIMP2, DDX21, LDHA, CCNA2, IFRD1, TCP1, NPM1, PSMA1, PCNA, NCBP1, RPL34, HSPD1, SRSF7, PRDX3, HNRNPC, CYC1, CDK2, NOP16, GSPT1, PSMA2, MRPL9, MCM6, GLO1, PSMD14, UBE2L3, RPS3, CANX, CCT7, CLNS1A, CCT4, PPM1G, RPL6, CDC45, PSMB2, STARD7, SNRPB2, HSP90AB1, PSMD1, CDC20, RSL1D1, SRM,

No.	Pathway	Leading-edge summary	Core enrichment genes
			SRPK1, CSTF2, PSMC6, UBA2, ACP1, CCT2, CBX3, RAN, PSMC4, SET, HNRNPA3, COX5A, EIF2S1, ILF2, DDX18, SNRPA, LSM7, EIF4A1, PSMA7, NHP2, GOT2, SLC25A3, EIF3J, FAM120A, C1QBP, CCT3, XPO1
15	UNFOLDED PROTEIN RESPONSE	tags=41%, list=18%, signal=34%	XBP1, DNAJB9, HERPUD1, SPCS3, PDIA6, DNAJC3, KDELR3, HSP90B1, HSPA5, FKBP14, SRPRB, EIF2AK3, EDEM1, DDIT4, HYOU1, SLC7A5, PDIA5, EXOSC9, WIPI1, NOLC1, SSR1, GOSR2, NOP56, CCL2, MTHFD2, DCP2, SLC30A5, SLC1A4, NPM1, EIF4A3, LSM1, SDAD1, SPCS1, EXOSC1, ASNS, DDX10, ATF4, ATF6, YIF1A, YWHAZ, CEBPB, CALR, PREB
16	UV RESPONSE (Up)	tags=36%, list=18%, signal=29%	HSPA13, E2F5, LYN, IL6, IL6ST, SOD2, BTG2, ARRB2, GLS, BCL2L11, FMO1, PTPRD, ICAM1, TFRC, BAK1, RASGRP1, RPN1, CASP3, PDLIM3, CA2, POLG2, CXCL2, POLR2H, TMBIM6, STIP1, DNAJB1, YKT6, ALAS1, BID, RAB27A, RRAD, ATP6V1C1, CHKA, PPIF, EIF2S3, PRKCD, DNAJA1, DDX21, AMD1, IRF1, ENO2, NAT1, SHOX2, NFKBIA, CDK2, PPT1, PLCL1, ASNS, OLFM1, GRPEL1, NXF1, CCNE1, NTRK3, EPCAM
17	ADIPOGENESIS	tags=44%, list=26%, signal=33%	C3, FABP4, UCP2, DNAJB9, PLIN2, ENPP2, COX7B, SPARCL1, IFNGR1, COL15A1, COL4A1, HSPB8, UQCRQ, DHRS7, SDHB, DDT, NDUFAB1, LAMA4, LPCAT3, PPP1R15B, ACADM, SLC1A5, IDH1, MGST3, COX6A1, SAMM50, AK2, ECH1, DLD, OMD, CD151, DRAM2, SORBS1, CMPK1, SUCLG1, CYP4B1, MRPL15, SCARB1, IMMT, REEP5, PFKFB3, PRDX3, MDH2, CYC1, ACLY, RAB34, NDUFA5, STOM, SLC5A6, GRPEL1, DLAT, PREB, AIFM1, ESYT1, DNAJC15, ORM1, COQ3, DGAT1, COX8A, UQCR11, ANGPT1, CPT2, GPX4, CHCHD10, GHITM, HIBCH, UQCR10, PTCD3, ANGPTL4, TALDO1, PGM1, DECR1, IDH3A, ITGA7, PHYH, UBQLN1, ATP1B3, CHUK, APOE, MYLK, UQCRC1, ETFB
18	KRAS SIGNALING (Down)	tags=46%, list=29%, signal=33%	CPB1, MTHFR, WNT16, HTR1B, EFHD1, SCGB1A1, CYP11B2, P2RX6, EDAR, TFF2, MYOT, BRDT, FSHB, CAPN9, PAX4, IL12B, CLDN16, KLHDC8A, DTNB, SNCB, NR6A1, YPEL1, GPRC5C, SPTBN2, EDN2, MYO15A, CALCB, TGFB2, CKM, SGK1, SCN10A, THNSL2, ADRA2C, FGF16, CLPS, FGF22, MYH7, SERPINA10, KRT15, PNMT, ABCB11, ARHGDIG, ATP4A, GPR3, SLC12A3, HSD11B2, ZBTB16, ATP6V1B1, GP2, GAMT, NTF3, COPZ2, YBX2, NGB, KCNE2, CYP39A1, ABCG4, PKP1, FGFR3, BMPR1B, DLK2, P2RY4, SLC30A3, TCF7L1, CELSR2, PROP1, EGF, SSTR4, MX1, RSAD2, HNF1A, EPHA5, CHRNG, PDE6B, SLC6A3, CAMK1D, ALOX12B, CNTFR, CLDN8, TEX15, GRID2, TFAP2B, CALML5, THRB, KRT1, NOS1, CD207
19	REACTIVE	tags=35%, list=16%, signal=29%	FTL, SOD2, PRDX4, PDLIM1, GLRX, NDUFA6, HHEX, PFKP,

No.	Pathway	Leading-edge summary	Core enrichment genes
	OXYGEN SPECIES PATHWAY		SRXN1, GCLM, GLRX2, MSRA, TXNRD1, PRNP, PRDX1, GSR
20	p53 PATHWAY	tags=41%, list=25%, signal=31%	IL1A, MDM2, UPP1, SLC7A11, PITPNC1, TGFB1, DRAM1, RB1, BTG2, HBEGF, MXD1, TM4SF1, CLCA2, F2R, DDIT4, LDHB, TPD52L1, BAK1, PTPRE, SPHK1, TGFA, S100A10, CASP1, ADA, RPS27L, CCNG1, SERTAD3, FUCA1, KRT17, SOCS1, SEC61A1, RRAD, TCN2, IER3, POLH, FAS, RGS16, SAT1, EPHA2, CDKN2A, PDGFA, SLC3A2, ANKRA2, PCNA, KIF13B, DNTTIP2, TPRKB, CGRRF1, PHLDA3, GM2A, ST14, STOM, WRAP73, RCHY1, RPS12, ITGB4, RNF19B, JUN, FOS, CDKN1A, SFN, CDKN2AIP, CCP110, CYFIP2, STEAP3, PLK3, ZMAT3, RAP2B, S100A4, AK1, NDRG1, NHLH2, ISCU, PRKAB1, HSPA4L, ABAT, EPHX1
21	ANDROGEN RESPONSE	tags=33%, list=14%, signal=29%	SEC24D, MERTK, DNAJB9, SPCS3, IQGAP2, UBE2J1, LMAN1, STEAP4, TMPRSS2, PMEPA1, PGM3, B2M, B4GALT1, CDK6, INPP4B, UAP1, ELOVL5, PDLIM5, ALDH1A3, FADS1, ABHD2, SMS, ELL2, NGLY1, SAT1, PTK2B, MYL12A, SCD, ITGAV, ACTN1, ADAMTS1, IDI1
22	PROTEIN SECRETION	tags=46%, list=27%, signal=34%	SEC24D, ARFGAP3, LMAN1, CTSC, CD63, AP3B1, SCRN1, CAV2, USO1, YKT6, COPB1, CLN5, RER1, VPS4B, ADAM10, GOSR2, M6PR, TMX1, BET1, ARCN1, SNAP23, RAB9A, COPB2, COPE, TSG101, STAM, PPT1, GLA, GOLGA4, NAPG, AP1G1, ATP1A1, SEC31A, LAMP2, TMED2, TMED10, MON2, RAB2A, RAB22A, ERGIC3, PAM, STX7, AP2M1
23	ANGIOGENESIS	tags=42%, list=14%, signal=36%	SPP1, POSTN, LUM, FSTL1, OLR1, TIMP1, CXCL6, SLCO2A1, NRP1, KCNJ8, FGFR1, PDGFA, LRPAP1, ITGAV, STC1
24	GLYCOLYSIS	tags=36%, list=23%, signal=29%	CXCR4, ELF3, MERTK, KDELR3, SPAG4, FUT8, GPC3, HSPA5, ME2, GLRX, CTH, B4GALT1, AKR1A1, PLOD2, DDIT4, GFPT1, NT5E, TGFA, SDC2, UGP2, P4HA1, IDH1, PFKP, HAX1, GOT1, PMM2, CDK1, PRPS1, GUSB, B4GALT4, DLD, IER3, PYGL, SLC25A13, LDHA, NDUFV3, COPB2, DEPDC1, ENO2, CHST1, GALE, AURKA, STC1, TPST1, PKP2, ISG20, CHST2, MDH2, CHPF, HS6ST2, PLOD1, B3GNT3, GMPPA, SRD5A3, EXT2, TGFBI, GNE, DCN, ARPP19, AGL, GMPPB, ALG1, IL13RA1, MET, BPNT1, BIK, SLC35A3, HMMR, CHPF2, ANGPTL4, TALDO1
25	E2F TARGETS	tags=38%, list=24%, signal=29%	PRDX4, CKS2, KPNA2, EXOSC8, NME1, TFRC, LBR, PLK1, RAD1, SMC4, CDK1, NOLC1, MELK, PRPS1, AK2, NOP56, MAD2L1, NBN, RAD51AP1, TRIP13, CDKN3, NAA38, IPO7, CDKN2A, MTHFD2, WEE1, DEPDC1, PCNA, CENPE, SPC25, BRCA1, BRCA2, AURKA, DONSON, LYAR, MYBL2, GSPT1, DDX39A, TMPO, SNRPB, MCM6, HUS1, STAG1, PDS5B, CCNE1, DLGAP5, CDKN1B, GINS4, SMC6, NUP205, SPC24, NUP107, TIPIN, RAD21, PRKDC, PHF5A, PRIM2,

No.	Pathway	Leading-edge summary	Core enrichment genes
			RPA2, CDKN1A, HMGB3, PSIP1, CCP110, DCTPP1, MLH1, HMMR, CDC20, TACC3, MKI67, ASF1B, CCNB2, GINS3, DNMT1
26	XENOBIOTIC METABOLISM	tags=23%, list=12%, signal=21%	TDO2, AQP9, KYNU, MAN1A1, UPP1, SAR1B, IL1R1, BCAT1, FBP1, VNN1, FMO1, DHRS7, SSR3, ELOVL5, PDLIM5, DDT, IRF8, PTGES3, SERPINE1, CA2, FMO3, CFB, TMEM176B, TMBIM6, SLC1A5, SLC35B1, SHMT2, ALAS1, IDH1, CYP2E1, ABCD2, PGD, ATP2A2, CDA, ECH1, NPC1, FBLN1, FAS, PDK4, MT2A, PAPSS2, HPRT1, EPHA2, ESR1, ENPEP
27	G2/M CHECKPOINT	tags=37%, list=23%, signal=29%	TGFB1, ODC1, CKS2, KPNA2, SLC38A1, SLC7A5, RBL1, LBR, PLK1, SMC4, NUP50, CDK1, NOLC1, KIF20B, CDC6, PRPF4B, BUB3, SNRPD1, MAD2L1, MT2A, CDKN3, HIF1A, AMD1, CCNA2, PBK, BCL3, CENPE, SRSF10, BRCA2, AURKA, CDC27, KIF23, MYBL2, GSPT1, DDX39A, WRN, TMPO, TPX2, NDC80, MCM6, HUS1, HSPA8, ORC5, STAG1, PDS5B, CDKN1B, TTK, BUB1, ATRX, RAD21, KIF15, DBF4, STIL, PRIM2, DTYMK, RPA2, CDC45, HMGB3, FBXO5, E2F4, ARID4A, KIF11, HMMR, CDC20, TACC3, RBM14, E2F3, MKI67, CCNB2
28	CHOLESTEROL HOMEOSTASIS	tags=33%, list=14%, signal=28%	PLAUR, NFIL3, TP53INP1, PLSCR1, CXCL16, ANXA5, GPX8, ANTXR2, LGMN, GUSB, STARD4, FADS2, TNFRSF12A, ECH1, CHKA, CLU, ETHE1, STX5, ACTG1, ALCAM, MAL2, SCD, IDI1
29	HYPOXIA	tags=34%, list=23%, signal=27%	CXCR4, SLC2A3, IL6, PDK1, PLIN2, ETS1, KDELR3, GPC3, PLAUR, NFIL3, TGM2, HSPA5, PLAC8, GLRX, TNFAIP3, TES, FBP1, ANXA2, DDIT4, HEXA, WSB1, SERPINE1, SDC2, UGP2, P4HA1, BGN, PFKP, IER3, ZFP36, MT2A, LDHA, ADM, PRDX5, SCARB1, KLF7, SDC4, ENO2, STC1, PFKFB3, ISG20, CHST2, PPARGC1A, CAV1, NAGK, GCNT2, PHKG1, TGFBI, CDKN1B, LXN, SULT2B1, DCN, VHL, JUN, PRKCA, JMJD6, IDS, FOS, CDKN1A, TGFB3, GPI, NEDD4L, BHLHE40, ANGPTL4, HS3ST1, S100A4
30	APICAL JUNCTION	tags=30%, list=19%, signal=25%	PTPRC, PECAM1, SKAP2, CDH3, FBN1, CLDN7, CLDN4, LAMC2, ICAM2, RHOF, RAC2, B4GALT1, MMP9, CD86, THY1, ICAM1, ZYX, ADAM9, VWF, ARHGEF6, ITGA3, MSN, CALB2, FSCN1, MMP2, ACTB, PIK3R3, VCAM1, CD209, AKT3, CD274, VCL, TNFRSF11B, CAP1, BMP1, ACTG1, NEXN, IRS1, CDH11, CD34, YWHAH, AMIGO2, ACTN1, MYL12B, ITGA9, SGCE, EPB41L2, LIMA1, ADAMTS5, CDH6, CDH1, TGFBI, SYK, JAM3, MYL9, CERCAM, ITGB4, GNAI2
31	MITOTIC SPINDLE	tags=39%, list=28%, signal=29%	DOCK2, ARHGAP29, NEDD9, RHOF, BCL2L11, PREX1, KNTC1, CCDC88A, PDLIM5, RASA2, DOCK4, FSCN1, SSH2, PLK1, ROCK1, SMC4, ECT2, NET1, CDK1, KIF20B, PCGF5, FGD4, VCL, PLEKHG2, LRPPRC, CNTRL, STK38L, AKAP13, ALMS1, CENPE, ARHGEF2, BRCA2, AURKA, CD2AP, CDC27, ARHGDIA, KIF23, RAB3GAP1,

No.	Pathway	Leading-edge summary	Core enrichment genes
			CEP192, EPB41L2, ARAP3, TPX2, NDC80, DLGAP5, FARP1, TTK, BUB1, RICTOR, ARHGAP4, KIF15, ARHGEF7, FBXO5, PKD2, CENPJ, KIF11, ATG4B, ABI1, PXN, CDC42BPA, CCNB2, CDC42EP1, TUBGCP5, RAPGEF6, NIN, MID1IP1, BIN1, MYH10, CDC42EP2, NCK1, ANLN, LMNB1, SORBS2, SPTBN1, KIF5B, BCAR1, CAPZB, SOS1
32	TGF-β β SIGNALING	tags=34%, list=18%, signal=28%	TGFB1, SLC20A1, SKIL, PMEPA1, RAB31, ENG, SERPINE1, ID1, THBS1, ID3, UBE2D3, HDAC1, SMURF2, ARID4B, PPP1CA, CDH1, ACVR1, IFNGR2
33	INTERFERON ALPHA RESPONSE	tags=34%, list=20%, signal=27%	SELL, SAMD9, B2M, CD74, PLSCR1, RIPK2, SAMD9L, CCRL2, CASP1, C1S, TMEM140, PSMA3, TDRD7, NCOA7, GBP4, IFITM2, LAMP3, GBP2, NMI, CASP8, IRF1, IL4R, ISG20, IFITM3, STAT2, IFI35, PARP9, IL15, PARP14, EIF2AK2, PARP12
34	PEROXISOME	tags=24%, list=14%, signal=20%	HSD17B11, SOD2, UGT2B17, ACSL5, ELOVL5, CRABP2, ITGB1BP1, RDH11, FADS1, IDH1, SEMA3C, ABCD2, ACSL1, STS, VPS4B, ECH1, SLC23A2, PRDX5, GSTK1, BCL10, YWHAH, ACSL4, IDI1, PRDX1
35	FATTY ACID METABOLISM	tags=24%, list=15%, signal=21%	TDO2, HSD17B11, ODC1, HSPH1, VNN1, FMO1, ACSL5, ELOVL5, RDH11, S100A10, CA2, PDHA1, ACADM, HSP90AA1, G0S2, OSTC, HADHB, IDH1, ACSL1, SMS, ECH1, DLD, LGALS1, CPT1A, NBN, PCBD1, LDHA, SUCLG1, UROS, ERP29, SUCLG2, ENO2, YWHAH, ACSL4, IDI1, PTPRG, MDH2
36	UV RESPONSE (Down)	tags=28%, list=16%, signal=24%	TFPI, PMP22, DAB2, EFEMP1, ANXA2, INPP4B, NRP1, MRPS31, PDLIM5, RASA2, HAS2, SERPINE1, TGFBR2, ID1, LAMC1, SDC2, CELF2, KIT, PTPRM, GRK5, FBLN5, PIK3R3, FYN, RBPMS, AKT3, PDGFRB, ATP2B1, IRS1, ADD3, ITGB3, SYNJ2, SRI, APBB2, LPAR1, CAV1, LTBP1, GCNT1, COL5A2
37	SPERMATOGENESI S	tags=19%, list=12%, signal=17%	TKTL1, GPR182, NEFH, BRAF, PCSK4, YBX2, CLVS1, PCSK1N, GAD1, THEG, SPATA6, CHRM4, SNAP91, ZPBP, ACTL7B, DNAJB8, HOXB1, CNIH2, TNP1, TUBA3C, CFTR, PACRG, NAA11, HSPA2, NOS1
38	MYOGENESIS	tags=32%, list=21%, signal=25%	MYF6, CAMK2B, NQO1, ANKRD2, TSC2, CAV3, TNNT1, MYOM1, ABLIM1, TCAP, ITGB5, CKM, MYL6B, HDAC5, MYL3, BDKRB2, APOD, MYH7, ATP6AP1, MYL2, KCNH1, MYBPC3, EPHB3, GAA, MAPRE3, REEP1, CRYAB, MYL7, TNNI1, CKMT2, MYH4, MB, PDE4DIP, PPP1R3C, EFS, MYL1, SGCA, COX6A2, SMTN, SORBS3, ACTN2, CHRNG, MYOG, FOXO4, ATP2A1, ERBB3, AKT2, COL1A1, SLN, LDB3, TNNT2, DES, FKBP1B, CKB, VIPR1, MYBPH, TNNC1, TNNT3, SGCG, DAPK2, NOS1, CD36
39	(Down)A REPAIR	tags=44%, list=32%, signal=30%	TAF13, PNP, HCLS1, DAD1, NME1, GTF2A2, ADA, GTF2B, SDCBP, POLR2H, PDE4B, POLR2K, TAF12, SEC61A1, CDA, POLH, TYMS,

No.	Pathway	Leading-edge summary	Core enrichment genes
			HPRT1, PCNA, COX17, CANT1, TSG101, GMPR2, SURF1, STX3, VPS37B, ELL, EIF1B, GPX4, RPA2, RRM2B, POLR2C, POLR2I, EDF1, TMED2, RALA, SRSF6, TK2, AK1, POLR2E, BCAP31, TAF6, ERCC1, RAD51, POLB, RAE1, RBX1, DCTN4, SF3A3, SNAPC5, CETN2, TAF10, CSTF3, POLR3C, BCAM, NT5C, NME4, POLD4, NUDT21, ERCC4
40	PI3K AKT mTOR SIGNALING	tags=30%, list=21%, signal=24%	CXCR4, PDK1, SLA, HSP90B1, IL2RG, IRAK4, RALB, DAPP1, PRKCB, RAC1, PIK3R3, CDK1, TBK1, GRB2, CAB39, UBE2D3, YWHAB, ARHGDIA, CDK2, PPP1CA, MAPKAP1, STAT2, CALR, RIT1, UBE2N, CDKN1B, MAPK8, NFKBIB, CDKN1A, SFN, ITPR2
41	APICAL SURFACE	tags=23%, list=9%, signal=21%	RHCG, LYN, PLAUR, IL2RG, B4GALT1, THY1, ATP6V0A4, MAL, IL2RB, ADAM10
42	Wnt β Catenin SIGNALING	tags=36%, list=20%, signal=29%	MYC, CCND2, WNT5B, HDAC5, JAG2, TP53, NCOR2, DVL2, HDAC11, NKD1, JAG1, KAT2A, WNT1, DLL1, FZD8
43	HEME METABOLISM	tags=34%, list=28%, signal=25%	C3, UCP2, CLIC2, CTSB, SLC7A11, BTG2, SEC14L1, MBOAT2, GYPC, TFRC, ATG4A, USP15, SDCBP, BMP2K, CA2, HBB, MGST3, ELL2, BPGM, GCLM, BACH1, FBXO34, MARK3, UROS, CAST, CDC27, ASNS, ARL2BP, PDZK1IP1, DCUN1D1, FBXO7, MPP1, LPIN2, NARF, AHSP, YPEL5, CDR2, ACP5, HEBP1, LAMP2, BSG, ENDOD1, SLC25A37, RNF19A, UROD, NFE2, TMCC2, SYNJ1, RAP1GAP, PPOX, NEK7, ICAM4, SLC11A2, CA1, SLC22A4, GYPB, XPO7, PICALM, NNT, CTNS, SNCA, EPB42, RIOK3
44	BILE ACID METABOLISM	tags=18%, list=12%, signal=16%	AQP9, HSD17B11, BMP6, ACSL5, DIO2, FADS1, ABCA9, ABCA6, IDH1, ABCD2, ACSL1, GCLM, FADS2, PNPLA8, NPC1, SLC23A2, PRDX5, NEDD4, GSTK1, RBP1
45	ESTROGEN RESPONSE EARLY	tags=18%, list=13%, signal=16%	XBP1, ELF3, IL6ST, CLDN7, RHOBTB3, TGM2, UGCG, MPPED2, B4GALT1, PRSS23, HSPB8, ELOVL2, TPD52L1, ELOVL5, MUC1, RAB31, RASGRP1, PODXL, ISG20L2, KRT13, SLC7A5, PDLIM3, ABHD2, CALB2, OLFML3, NRIP1, PPIF, MYBL1, PAPSS2, SCARB1, SLC1A4, SLC1A1, CHPT1, ADD3

Table S7. Complete GSVA differential pathway statistics.

No.	Pathway	logFC	Average expression	t statistic	Nominal P value	Adjusted P value	B statistic
1	IL-2 STAT5 SIGNALING	0.2596	5.795e-04	3.9165	3.939e-04	0.0053	-0.0102
2	Allograft Rejection	0.3375	-0.007	3.8566	4.678e-04	0.0053	-0.1698
3	mTORC1 SIGNALING	0.3007	0.0209	3.8485	4.788e-04	0.0053	-0.1914
4	Complement	0.2905	0.0035	3.8402	4.904e-04	0.0053	-0.2135
5	Reactive Oxygen Species Pathway	0.2602	-8.018e-04	3.7891	5.675e-04	0.0053	-0.3489
6	Inflammatory Response	0.304	-0.0071	3.7509	6.327e-04	0.0053	-0.4495
7	UV Response (Up)	0.2027	0.0081	3.5907	9.946e-04	0.0071	-0.8673
8	IL-6 JAK STAT3 SIGNALING	0.3304	0.0069	3.4681	0.0014	0.0087	-1.1814
9	Oxidative Phosphorylation	0.3047	0.0158	3.3715	0.0018	0.0101	-1.4255
10	Glycolysis	0.1852	0.009	3.2712	0.0024	0.012	-1.6752
11	Apoptosis	0.2223	0.0102	3.0462	0.0044	0.0199	-2.2207
12	Adipogenesis	0.1894	0.0196	2.9261	0.006	0.0233	-2.5028
13	Unfolded Protein Response	0.2303	0.0083	2.92	0.0061	0.0233	-2.5171
14	Androgen Response	0.1814	0.0244	2.7567	0.0092	0.0312	-2.8893
15	MYC TARGETS V1	0.21	0.0161	2.7492	0.0094	0.0312	-2.9061
16	TNFα SIGNALING VIA NF-κB	0.2285	-0.0094	2.6131	0.0131	0.0405	-3.2054
17	KRAS SIGNALING UP	0.1988	0.0047	2.5926	0.0138	0.0405	-3.2498
18	Wnt/β-Catenin SIGNALING	-0.1439	-0.0035	-2.5158	0.0166	0.0454	-3.4134
19	p53 PATHWAY	0.1502	0.0045	2.4989	0.0173	0.0454	-3.4489
20	Interferon-γ Response	0.2669	0.0254	2.4686	0.0186	0.0464	-3.5122
21	Xenobiotic Metabolism	0.1086	0.0101	2.1744	0.0365	0.0868	-4.0981
22	Protein Secretion	0.1986	0.033	2.1088	0.0421	0.0958	-4.2211

No.	Pathway	logFC	Average expression	t statistic	Nominal P value	Adjusted P value	B statistic
23	Coagulation	0.1481	0.0106	1.9427	0.0601	0.1306	-4.5195
24	KRAS SIGNALING DN	-0.1313	-0.0055	-1.9018	0.0654	0.1362	-4.59
25	Cholesterol Homeostasis	0.1379	0.0027	1.8683	0.07	0.1401	-4.6469
26	TGF-β SIGNALING	0.1408	0.0055	1.812	0.0785	0.1496	-4.7405
27	Epithelial-Mesenchymal Transition	0.2041	0.0014	1.7979	0.0808	0.1496	-4.7637
28	Hypoxia	0.0859	-2.796e-04	1.5611	0.1274	0.2276	-5.1288
29	Peroxisome	0.0855	0.0144	1.4379	0.1593	0.2673	-5.301
30	Angiogenesis	0.1706	0.0119	1.434	0.1604	0.2673	-5.3063
31	PI3K AKT mTOR SIGNALING	0.0789	0.0119	1.3949	0.1718	0.2771	-5.3582
32	Fatty Acid Metabolism	0.0948	0.0073	1.3488	0.186	0.2906	-5.4178
33	DNA Repair	0.091	0.0176	1.2664	0.2137	0.3237	-5.5198
34	Apical Junction	0.0827	-1.580e-04	1.2334	0.2256	0.3317	-5.559
35	Myogenesis	-0.0798	-0.0026	-1.1626	0.2528	0.3612	-5.64
36	Spermatogenesis	-0.0485	-0.0031	-1.0168	0.3162	0.4391	-5.7926
37	G2/M CHECKPOINT	0.0864	0.0157	0.9635	0.3419	0.4572	-5.8436
38	Mitotic Spindle	0.0691	0.0161	0.9522	0.3475	0.4572	-5.8541
39	Heme Metabolism	0.0491	0.0045	0.9278	0.3598	0.4613	-5.8763
40	E2F TARGETS	0.0907	0.0284	0.9064	0.3709	0.4636	-5.8954
41	Interferon-α Response	0.1024	0.0084	0.8362	0.4087	0.4984	-5.9548
42	Pancreas β Cells	-0.0615	-0.0192	-0.6996	0.4888	0.5819	-6.0574
43	HEDGEHOG SIGNALING	-0.0536	0.0134	-0.6442	0.5236	0.6089	-6.0939
44	UV Response (Down)	0.0392	0.0118	0.484	0.6314	0.7064	-6.1829
45	MYC TARGETS V2	0.0423	0.0148	0.4778	0.6357	0.7064	-6.1858
46	Apical Surface	-0.0221	-5.191e-04	-0.3288	0.7443	0.809	-6.2453

No.	Pathway	logFC	Average expression	t statistic	Nominal P value	Adjusted P value	B statistic
47	NOTCH SIGNALING	-0.0148	-3.717e-04	-0.2718	0.7874	0.8377	-6.2623
48	Estrogen Response (Early)	0.0103	-0.0029	0.1817	0.8569	0.8926	-6.2825
49	Bile Acid Metabolism	-0.0047	0.0073	-0.0865	0.9316	0.9506	-6.2952
50	Estrogen Response (Late)	0.0012	0.002	0.0207	0.9836	0.9836	-6.2987

Table S8. Internal validation of BRAF, TRPV1, and the combined model as exploratory candidate features.

Model	Features	Apparent AUC (95% CI)	LOOCV AUC (95% CI)	Repeated 5-fold CV mean AUC ± SD
BRAF	BRAF	0.808 (0.631–0.985)	0.774 (0.587–0.961)	0.789 ± 0.022
TRPV1	TRPV1	0.793 (0.611–0.975)	0.721 (0.518–0.925)	0.761 ± 0.017
BRAF + TRPV1	BRAF + TRPV1	0.856 (0.693–1.000)	0.745 (0.547–0.943)	0.776 ± 0.034

Apparent AUC was calculated in the integrated cohort. LOOCV, leave-one-out cross-validation; CV, cross-validation; SD, standard deviation. These results indicate exploratory internal discriminatory performance and should not be interpreted as validated clinical diagnostic utility.

Table S9. Immune-cell comparisons between peri-implantitis and healthy control samples with multiple-testing correction.

Immune cell type	Mean in peri-implantitis	Mean in control	P value	Adjusted P value	Direction
Monocytes	0.0573	0.1006	0.0031	0.0677	Lower in peri-implantitis
T cells CD8	0.0939	0.1699	0.0070	0.0770	Lower in peri-implantitis
B cells memory	0.0470	0.0296	0.0825	0.4556	Higher in peri-implantitis
Neutrophils	0.0477	0.0178	0.0966	0.4556	Higher in peri-implantitis
NK cells activated	0.0280	0.0443	0.1157	0.4556	Lower in peri-implantitis
Plasma cells	0.2318	0.1771	0.1303	0.4556	Higher in peri-implantitis
T cells CD4	0.1073	0.0752	0.1670	0.4556	Higher in

memory resting					peri-implantitis
Dendritic cells activated	0.0326	0.0215	0.1808	0.4556	Higher in peri-implantitis
B cells naive	0.0111	0.0210	0.2182	0.4556	Lower in peri-implantitis
Macrophages M0	0.0351	0.0464	0.2230	0.4556	Lower in peri-implantitis
Mast cells activated	0.1533	0.1162	0.2278	0.4556	Higher in peri-implantitis
T cells CD4 memory activated	0.0032	0.0131	0.3396	0.5968	No clear difference
T cells follicular helper	0.0235	0.0220	0.3641	0.5968	Lower in peri-implantitis
Mast cells resting	0.0001	0.0140	0.4069	0.5968	No clear difference
T cells CD4 naive	0.0013	0.0038	0.4069	0.5968	No clear difference
Macrophages M1	0.0266	0.0338	0.4423	0.6081	Lower in peri-implantitis
Dendritic cells resting	0.0077	0.0042	0.4764	0.6165	No clear difference
T cells regulatory (Tregs)	0.0027	0.0186	0.5221	0.6381	No clear difference
Macrophages M2	0.0779	0.0644	0.7092	0.8212	Lower in peri-implantitis
NK cells resting	0.0108	0.0062	0.8706	0.9577	Lower in peri-implantitis
Eosinophils	0.0013	0.0002	0.9602	1.0000	No clear difference
T cells gamma delta	0.0000	0.0000	1.0000	1.0000	No clear difference

P values were calculated using the Wilcoxon rank-sum test. Adjusted P values were calculated using Benjamini–Hochberg correction across the 22 LM22-defined immune-cell types.
